# Electrochemical and Spectroelectrochemical Studies on the Reactivity of Perimidine–Carbazole–Thiophene Monomers towards the Formation of Multidimensional Macromolecules versus Stable π-Dimeric States

**DOI:** 10.3390/ma14092167

**Published:** 2021-04-23

**Authors:** Malgorzata Czichy, Patryk Janasik, Pawel Wagner, David L. Officer, Mieczyslaw Lapkowski

**Affiliations:** 1 Faculty of Chemistry, Silesian University of Technology, M. Strzody 9, 44-100 Gliwice, Poland; Patryk.Janasik@polsl.pl (P.J.); Mieczyslaw.Lapkowski@polsl.pl (M.L.); 2 ARC Centre of Excellence for Electromaterials Science and the Intelligent Polymer Research Institute, University of Wollongong, Wollongong, NSW 2519, Australia; pawel@uow.edu.au (P.W.); davido@uow.edu.au (D.L.O.); 3 Centre of Polymer and Carbon Materials, Polish Academy of Sciences, 34 Curie-Sklodowska Str., 41-819 Zabrze, Poland

**Keywords:** perimidine, carbazole, 3-alkoxythiophene, multi-functionalized monomer

## Abstract

During research on cross-linked conducting polymers, double-functionalized monomers were synthesized. Two subunits potentially able to undergo oxidative coupling were used—perimidine and, respectively, carbazole, 3,6-di(hexylthiophene)carbazole or 3,6-di(decyloxythiophene)carbazole; alkyl and alkoxy chains as groups supporting molecular ordering and 14*H*-benzo[4,5]isoquinone[2,1-a]perimidin-14-one segment promoting CH⋯O interactions and π–π stacking. Electrochemical, spectroelectrochemical, and density functional theory (DFT) studies have shown that potential-controlled oxidation enables polarization of a specific monomer subunit, thus allowing for simultaneous coupling via perimidine and/or carbazole, but mainly leading to dimer formation. The reason for this was the considerable stability of the dicationic and tetracationic π-dimers over covalent bonding. In the case of perimidine-3,6-di(hexylthiophene)carbazole, the polymer was not obtained due to the steric hindrance of the alkyl substituents preventing the coupling of the monomer radical cations. The only linear π-conjugated polymer was obtained through di(decyloxythiophene)carbazole segment from perimidine-di(decyloxythiophene)-carbazole precursor. Due to the significant difference in potentials between subsequent oxidation states of monomer, it was impossible to polarize the entire molecule, so that both directions of coupling could be equally favored. Subsequent oxidation of this polymer to polarize the side perimidine groups did not allow further crosslinking, because rather the π–π interactions between these perimidine segments dominate in the solid product.

## 1. Introduction

Traditional π-conjugated polymers (CPs) that are chain-like macromolecules with one-dimensional (1D) charge transport along conjugated backbones have received considerable attention for possible application in various optoelectronic devices. Extending π-conjugation in CPs from 1D to 2D (two-dimensional) can not only give significantly enhanced charge transport [1] but also may induce other favorable properties, such as excellent flexibility [2,3], isotropic properties [4], etc. However, electronic properties are also closely related to molecular chain orientation resulting from the formation of covalent linking and noncovalent intramolecular and intermolecular interactions, and so ordering on even the level of three dimensions (3D). Generally, two-, three- and multidimensional molecular network can be organized based on types of monomer linking (covalently, coordinating and/or supramolecular), where can indicate such as: covalent organic frameworks (COF) [5,6], coordination polymers [7], metal-organic frameworks (MOF) [8] and supramolecular polymers [9,10,11]. Supramolecular structures are self-assembled small molecules held together by reversible noncovalent interactions, such as hydrogen bonds [12,13], coordination [14], π–π stacking [15,16], and host-guest interactions [17,18], etc. It is noteworthy that supramolecular polymers do not comply with classical Carothers’ definition of a polymer as a single macromolecule but rather the idea of polymers as colloidal aggregates. Morphology of π-conjugated one-dimensional polymer is also determined by self-assembly processes and so molecular packing and orientation during crystallization initiated by: π–π interactions between aromatic rings e.g., in poly(3-hexylthiophene) (P3HT) [19], oligo(pyrrole)s [20,21], directional and strong H-bonding interactions [22] and coordination bonds [23]. Different types of covalent bonding with accompanying long-range molecular interactions are present in low-band polymers such as multidimensional donor–acceptor systems [24,25] and conjugated ladder polymers (cLP) [26,27,28], where covalent bonds are formed through polymerization while noncovalent bonds can be generated simultaneously because of the dynamic and spontaneous nature of the noncovalent bonds [29]. Another strategy intended to increase planarity is to hamper rotations between the neighboring units by connecting them with covalent bonds or via noncovalent interactions. Insertion of backbone substituents, such as side chains or fused aromatic rings, also plays a role in lamellar stacking orientation [30,31].

In designing multidimensional structures, important is the monomer/monomers structure adjustment to achieve multidirectional propagation in one or more synthetic steps. For example, covalently non-conjugated cross-linked polymers (e.g., hydrogels) are usually prepared by bringing together small multi-functional molecules such as e.g., metacylates [32]. Crosslinking may be achieved by the reaction of two chemical groups on two different molecules, which can be initiated by catalysts, by photo-polymerization or by radiation crosslinking. In turn, three-dimensional π-conjugated architectures can be realized by using polyfunctional monomers or co-monomers (copolymerization) either by chemical or electrochemical oxidative coupling. In particularly potential-controlled redox processes are more effective than chemical ones because they provide control of the oxidation state, for this reason, the electrochemical polymerization [33,34]and crosslinking of side-units are successfully used [35,36]. The cross-linked structures were produced by oxidation of electropolymerizable groups, where coupling process via the 3, 6-position of carbazole unit or 2, 5-position of thiophene unit forming conjugated polymer network [37]. An electropolymerizable 3D-π-conjugated system with 3,4-ethylenedioxythiophene (EDOT) end-groups attached to a bithiophene core twisted by ca. 90 degrees used as a precursor of the electroactive conjugated network [38]. A branched electrochromic polymer was fabricated electrochemically from 4-(N-carbazolyl)triphenylamine precursors [39], multi-site monomers incorporating three or four carbazole [40,41] or thiophene [42,43] units into the branched backbone of the monomer. However, two-dimensional π-conjugated networks are more common with co-presence with coordination bonds as in regioregular polythiophene–porphyrin copolymers [44] and with long-range interaction in the third dimension—π–π-stacking [45,46,47] or H-bonding [48].

**Scheme 1:**
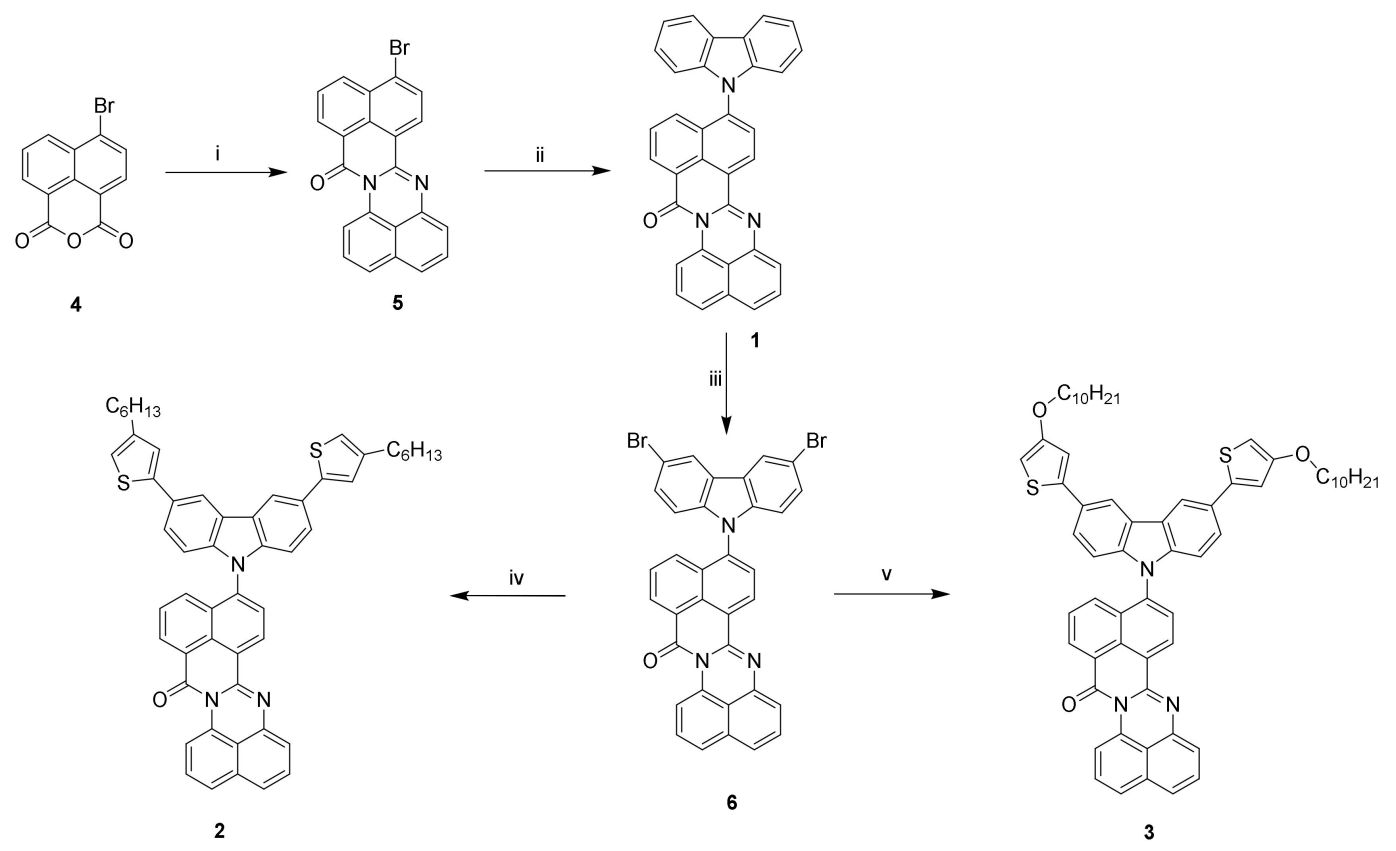
Synthesis of compounds **1**–**3**. (i) 1,8-diaminonaphthalene, methanesulphonic acid, NMP, reflux, 50%; (ii) carbazole, toluene, Pd(dppf)_2_Cl_2_, *t*-BuONa, 65%; (iii) N-bromosuccinimide (NBS), CHCl_3_, 93%; (iv) 4-hexyl-2-thienboronic acid sodium salt, Pd(PPh_3_)_4_, 1,2-dimethoxyethane (DME), K_2_CO_3,_ 25%; (v) 4-decyloxy-2-thienboronic acid, Pd(PPh_3_)_4_, DME, K_2_CO_3_, 90%.

We proposed monomers as carbazole derivatives—3,6-unsubstituted one (**1**) and 3,6-substituted by hexyl- (**2**) or decyloxythiophene rings (**3**) and each being substituted by 14H-benz[4,5]isoquino[2,1-a]perimidin-14-one unit (Scheme 1). Perimidine is characterized by both π-excessive and π-deficient arrangements, where lone pairs of nitrogen atoms transfer their electron density to naphthalene ring, which increases the occurrence of both electrophilic and nucleophilic reactions, acid-mediated *peri*-annulation e.g., to benzo[*gh*]perimidines [49], oxidative and electrooxidative coupling [50] or coordination [51]. Similar carbazole-perimidine derivatives have already been tested as donor–acceptor compounds for electronics as presented in [52,53]; however, electrochemical studies did not provide conclusive information about perimidine reactivity. Therefore, it seems that the application of discussed precursors may favor the formation of multidimensional structures, while the proposed study will contribute to broadening the knowledge in the field of the structural design of monomers and their electrochemical, potential-controlled polymerization.

## 2. Materials and Methods

### 2.1. Materials

All reagents for synthesis and measurements were purchased from Sigma Aldrich vendor (St. Louis, MO, USA). 4-Bromo-1,8-naphthyl (**4**) (95%), 1,8-diaminonaphthalene (99%), methanesulphonic acid (99%), carbazole (95%), toluene (anhydrous 99.8%), [1,1′-bis(diphenylphosphino)ferrocene]dichloropalladium(II) (Pd(dppf)_2_Cl_2_, 99%), sodium tert-butoxide (*t*-BuONa, 97%), N-bromosuccinimide (NBS, 99%), chloroform (CHCl_3_, anhydrous 99%), 4-hexyl-2-thienboronic acid sodium salt (97%), tetrakis(triphenylphosphine)palladium(0) (Pd(PPh_3_)_4,_ 99%), 1,2-dimethoxyethane (DME, anhydrous 99.5%), potassium carbonate (K_2_CO_3,_ 99.995%), 4-decyloxy-2-thienboronic acid (97%), tetrabutylammonium tetrafluoroborate (Bu_4_NBF_4_, 99.0%) dried under vacuum; dichloromethane (DCM, for HPLC, 99.8%), ferrocene (Fc, 98%) and argon (6.0, SIAD Group) were used.

### 2.2. Synthesis of Monomers

11-Bromobenzo[4,5]isoquino[2,1-a]perimidin-14-one (**5**) was obtained by the condensation reaction of 4-bromo-1,8-naphthyl (**4**) anhydride with 1,8-diaminonaphthalene. The bromine in this compound was displaced with carbazole in a modified Buchwald reaction to give derivative **1**. The compound **1** was obtained as a mixture of two isomers: 11- and 10-bromoderivative in ratio 1:1, which could not be separated. Compounds **2** and **6** [53], 4-decyloxy-2-thienylboronic acid [54], 4-hexyl-2-thienyl sodium boronate [55] were synthesized according to previous reports. Bromination of **1** gave the 3,6-dibromo derivative (**6**) which was reacted by a conventional Suzuki reaction with 4-hexyl-2-thienylboronic acid sodium salt or 4-decyloxy-2-thienylboronic acid to give **2** and **3** derivatives. Suzuki coupling general procedure: **1** (280 mg, 4.4 × 10^−4^ mol) and boronic acid (or the sodium salt) (1.74 × 10^−3^ mol) were dissolved in 1,2-dimethoxyethane (DME, 10 mL) and brought to boiling. The aqueous solution of potassium carbonate (1M, 5 mL) was added followed by Pd(PPh_3_)_4_ (37 mg, 4% mol per site). The resulting mixture was refluxed overnight under argon. After cooling, dichloromethane (50 mL) was added. The organic phase was separated, dried over magnesium sulfate and evaporated to dryness under reduced pressure at 50 °C. The remaining was dissolved in dichloromethane (~20 mL) and filtered through a pad of silica. The solvent was evaporated to dryness under reduced pressure at 50 °C. The remaining was dissolved in a minimal amount of dichloromethane and precipitated with methanol. The resulting solid was filtered off, washed with methanol then dried. **1**: Yield: 62%; ^1^H NMR (CDCl_3_, 400 MHz) δ_H_: 9.06 (d, 1H, *J* = 8.1 Hz), 8.93 (dd, 1H, *J* = 1.5 and 7.5 Hz), 8.78 (d, 1H, *J* = 7.8 Hz), 8.70–8.63 (m, 3H), 8.26–8.20 (m, 4H), 7.90 (d, 2H, *J* = 8.1 Hz), 7.74–7.31 (m, 22 H), 7.10–7.04 (m, 4H). **2**: Yield: 25%; ^1^H NMR (CDCl_3_, 400 MHz) δ_H_: 9.20 (d, 1H, *J* = 7.8 Hz), 8.99 (dd, 1H, *J* = 1.2 and 7.4 Hz), 8.75–8.68 (m, 5H), 8.61–8.55 (5H), 8.92–7.81 (m, 16H), 7.35–7.25 (m, 10H), 7.04 (d, 1H, *J* = 1.0 Hz), 7.02 (d, 1H, *J* = 1.0 Hz), 7.01 (d, 1H, *J* = 1.0 Hz), 6.99 (d, 1H, *J* = 1.0 Hz), 2.68–2.56 (m, 8H), 1.71–1.58 (m, 8H), 1.39–1.20 (m, 24H), 0.89–0.76 (m, 12H); HRMS: Found: [M+H]^+^ 818.3254, molecular formula C_54_H_48_N_3_OS_2_ requires [M + H]^+^ 818.3233. **3**: Yield: 90%; ^1^H NMR (CDCl_3_, 400 MHz) δ_H_: 9.28 (d, 1H, *J* = 8.1 Hz), 9.15 (dd, 1H, *J* = 0.6 and 4.3 Hz), 8.81 (d, 1H, *J* = 7.8 Hz), 8.68 (dd, 1H, *J* = 1.2 and 7.3 Hz), 8.65 (dd, 1H, *J* = 0.6 and 8.0 Hz), 8.64 (dd, 1H, *J* = 0.6 and 8.0 Hz), 8.04–8.00 (m, 2H), 7.97 (d, 1H, *J* = 8.0 Hz), 7.93 (d, 1H, *J* = 7.7 Hz), 7.90–7.88 (m, 2H), 7.73 (dd, 1H, *J* = 1.1 and 8.4 Hz), 7.70–7.68 (m, 2H), 7.63 (dd, 1H, *J* = 7.2 and 8.0 Hz), 7.54–7.48 (m, 2H), 7.43–7.33 (m, 14H), 7.11–7.06 (m, 4H), 7.04–7.02 (m, 2H), 6.43 (d, 1H, *J* = 1.6 Hz), 6.42 (d, 1H, *J* = 1.6 Hz), 6.36 (d, 1H, *J* = 1.6 Hz), 6.35 (d, 1H, *J* = 1.6 Hz), 4.07–3.97 (m, 8H), 1.88–1.75 (m, 8H), 1.51–1.22 (m, 56 H), 0.92–0.84 (m, 12H); HRMS: Found: [M + H]^+^ 962.4401, molecular formula C_62_H_64_N_3_O_3_S_2_ requires [M + H]^+^ 962.4384.

### 2.3. Characterization Methods

The *NMR spectra* were recorded on Bruker Avance 400 spectrometer with tetrametylosilan (TMS) as an internal standard. The HRMS spectra were collected on Waters Xevo QTOF mass spectrometer. *DFT/TD-DFT calculations* were carried out with B3LYP [56] hybrid functional combined with 6-31G(d) basis set. All calculations in this work were performed using the ORCA 4.1.1. package quantum chemistry programs [57]. Input files and molecular orbital plots were prepared with Gabedit 2.4.7 software [58]. Optical properties were investigated employing TD-DFT [59] calculations at the B3LYP level in combination with the 6-31G(d) basis set. To make calculations easier, it was decided to replace long alkyl chains with ethyl chains. *Cyclic voltammograms* were done by using CH Instruments Electrochemical Analyzer model 620. The experimental setup was in the single-cell and three electrode: 2 mm^2^—platinum disk (working electrode), platinum coil (counter electrode), and Ag|Ag^+^ pseudo-reference electrode calibrated with ferrocene|ferrocenium Fc|Fc^+^ couple as the internal standard. Monomer concentration was 1.0 mM in DCM in the presence of 0.1 M Bu_4_NBF_4_ deoxygenated by flushing with argon. HOMO (Highest Occupied Molecular Orbital) and LUMO (Lowest Unoccupied Molecular Orbital) energies were graphically determined from the onset potential ($E_{on}$) of the first oxidation and reduction using the Trasatti equations for non-aqueous electrolytes:$\;\mathrm{HOMO}\;\left(\mathrm{LUMO}\right)\left(\mathrm{eV}\right)=-5.1-\left(E_{\mathrm{on}}-E_{\mathrm{Fc}|\mathrm{Fc}+}\right)$, where $E_{\mathrm{Fc}|\mathrm{Fc}+}$ is a half-wave potential of Fc|Fc^+^ [60]. *UV-Vis-NIR spectroelectrochemical measurement* was performed using Agilent/HP 8453 UV-Visible Spectrophotometer G1103A. The measuring system was a quartz cuvette with an optical length equal to 2 mm with platinum mesh as working electrode, Ag|Ag^+^ as pseudo-reference electrode and platinum coil as counter electrode. The concentration of the sample was equal to 0.25 mM in each case. For spectroelectrochemical measurement of deposited **p(3)** was used ITO (Indium Tin Oxide)/quartz electrode (20 ± 5 ohm/sq, Praezisions Glas & Optic GmbH, Iserlohn, Germany) as a working electrode.

## 3. Results and Discussion

### 3.1. Quantum Chemical Calculations

Frontier molecular orbitals and spin densities are visualized in Tables S2 and S3. The energy of the HOMO orbital decreases in the monomer series **1**, **2**, **3** and is as follows −4.85, −4.40, −4.25 eV, respectively. Orbital HOMO is localized only on the perimidine unit in the case of **1**, but on both thiophenes rings in the structure of **2** and **3**. The energy of HOMO-1 is higher than HOMO by only 0.08 eV and is for **2** and **3**, respectively, −4.48 and −4.33 eV, while the orbital itself is shifted on the dithiophenecarbazole with a predominance of thiophene rings alone. The cases, where the molecular orbital extends on both segments of the monomer (i.e., perimidine and dithiophenocarbazole) are HOMO-1 for **1** and HOMO-2 for **2** and **3**. This is important that orbital HOMO-1 of compound **1** is distributed between perimidine and carbazole, and its energy reaches the value of −5.54 eV, so it is relatively low, such that the two subunits of **1** can be relatively easily polarized with the same potential. In turn, the energy of the second-to-highest occupied molecular orbital HOMO-2 amounts to −4.94 and −4.93 eV for **2** and **3**, respectively and its localization to the perimidine segment and both thiophenes is observed, while for **1** is already −5.79 and its location is completely shifted to the carbazole region. Generally, the first and second oxidation peaks of **2** and **3** should be separated by a low potential barrier and correspond to the generation of the radical cation and its transformation to the diradical dication, observed in the range from 0.3 to 0.65 V.

LUMO and LUMO+1 orbitals are located on the whole 14H-benz[4,5]isoquino[2,1-a]perimidin-14-one fragment for all monomers, but their residual presence also appears on carbazole in the case of LUMO+1 of compounds **2** and **3**. LUMO energy for **2** and **3** is similar in value, i.e., −2.81 and −2.80 eV, respectively, and is slightly smaller than that for **1** (−2.68 eV). However, the energy of the LUMO+1 orbital is already much higher and reaches values of −1.32, −1.44 and −1.43 eV for **1**, **2**, and **3**, respectively.

**Figure 1:**
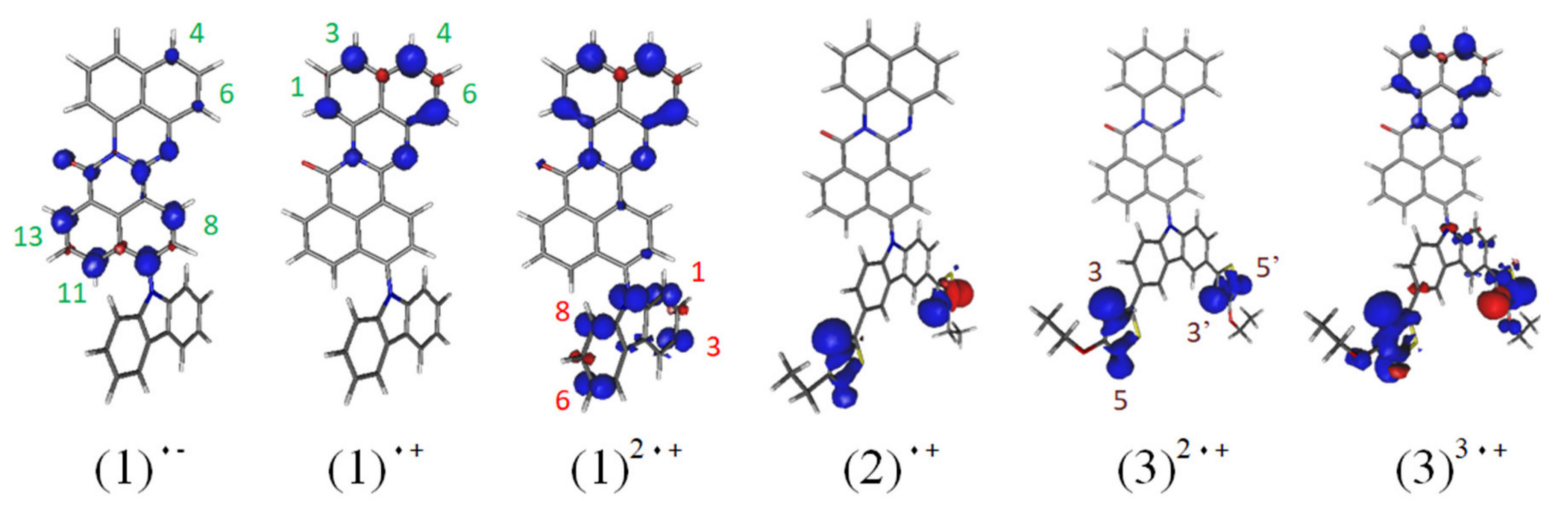
Spin density located at positions of carbazole (*red*), thiophenes (*brown*) and 14H-benz[4,5]isoquino[2,1-a]perimidin-14-one unit (*green*) of selected oxidized and reduced states of 1–3 moieties—calculated at B3LYP/6-31G(d)/CPCM(DCM), isovalue equal to 0.005 e^−^/au ^3^ in each case.

Spin densities are located only on the perimidine ring at positions 1, 3, 4, and 6 in the case of the radical cationic state of **1**, then it is additionally present on the carbazole segment for diradical dicationic and triradical tricationic states. The rotation of both subunits of **1** occurs in the tricationic triradical and indicates that this state can be regarded as a radical cation located on the perimidine, isolated from the diradical dications on carbazole segment. In turn, for radical cation and diradical dication of **2** and **3**, the spin density only occurs on the thiophene (3 and 5-positions), and additionally on carbazole (1, 3, 6, 8-positions) and perimidine (1, 3, 4, 6-positions) for these triradical trications. The spin density of all monomer radical anions increased in the entire segment of 14H-benz[4,5]isoquino[2,1-*a*]perimidin-14-one (4, 6, 8, 11, 13-positions) and additionally in 3-position in case of these diradical dianions (Figure 1, Table S3). Geometrical optimization of neutral and charged monomers confirms the localization of thiophene and carbazole group in one plane, while perimidine is perpendicular towards them, which indicates the break of π-conjugation between perimidine and carbazole segment in compounds **2** and **3**, too.

**Figure 2:**
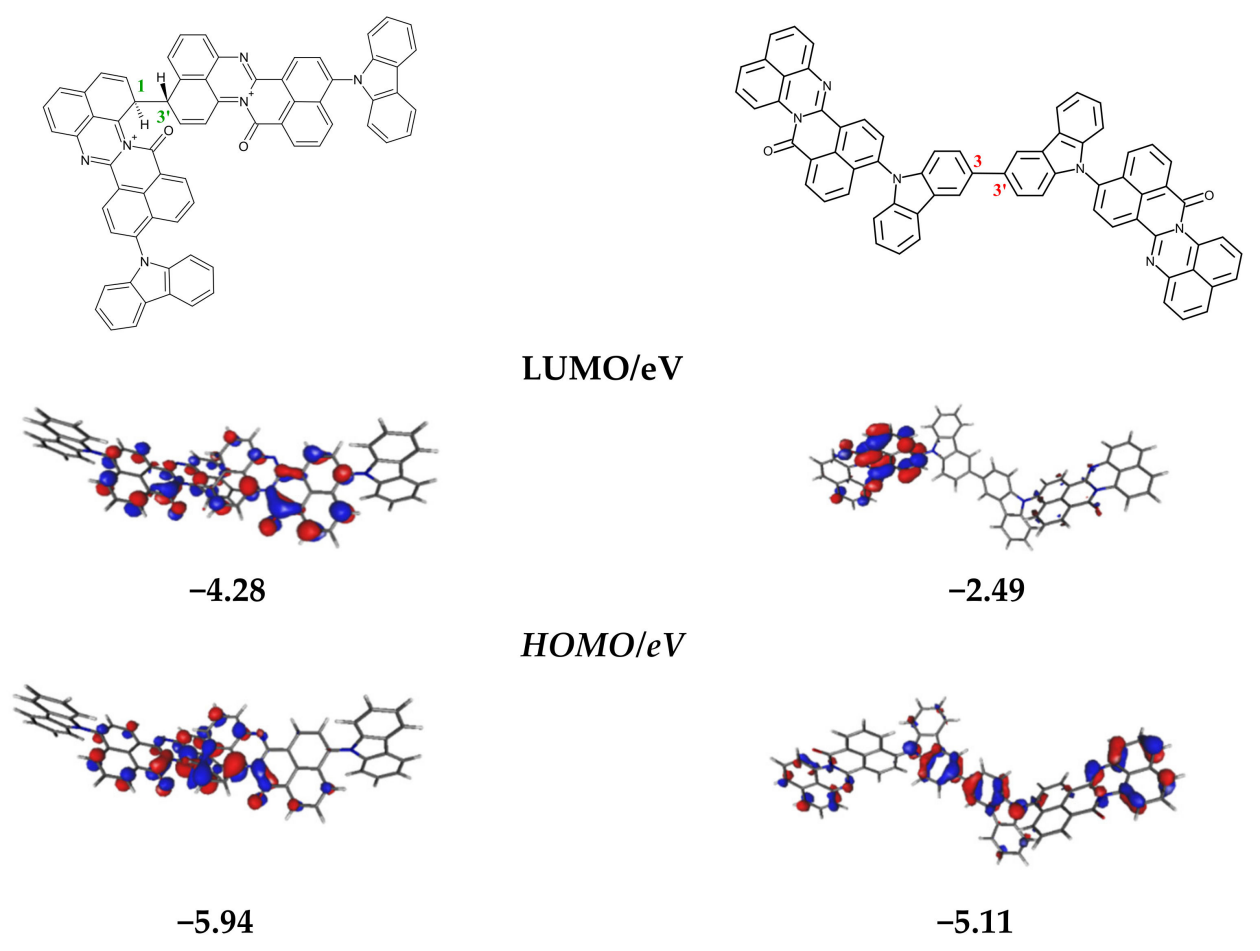

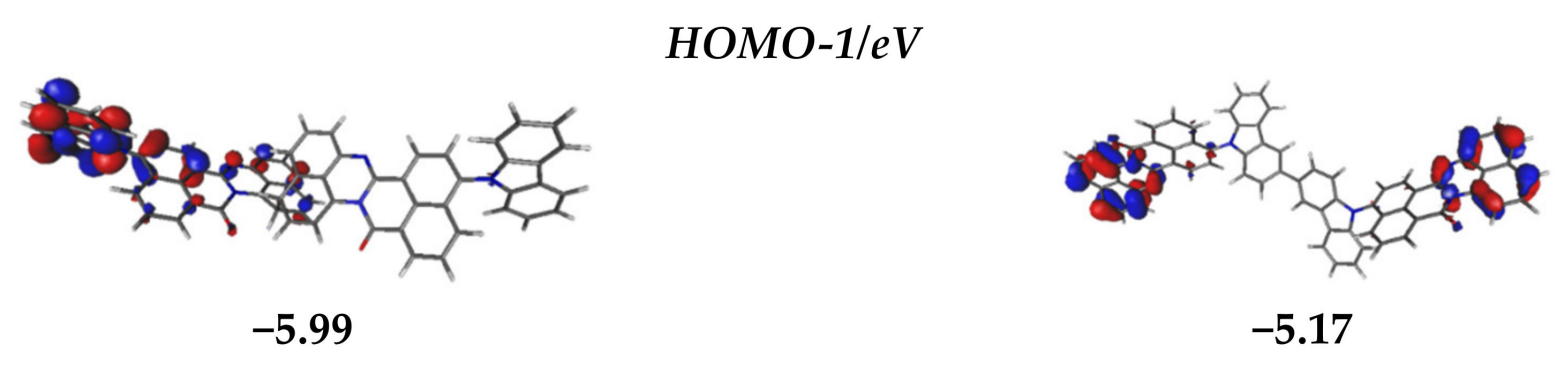
The shape of frontier orbitals of protonated perimidine–perimidine dimer of **1** (**left**) and neutral carbazole–carbazole dimer of **1** (**right**) calculated at B3LYP/6-31G(d)/CPCM(DCM). The isovalue is equal to 0.03 e^−^/au^3^ in each case.

There were also performed calculations for dimers, which may arise as a reaction product between radical cation or diradical dication states of compound **1**. These are protonated and deprotonated (neutral) dimers (Figure 2 and Table S4) and π-dimers (Table S6). In the case of covalently bonded dimers, several bonding possibilities have been assumed, where a new bond exists between the perimidine–perimidine, perimidine–carbazole, carbazole–carbazole segments. HOMO energies of the neutral dimers are similar in value to this energy of the precursor. In turn, the LUMO energies of protonated dimers assume similar values in the range −3.87–(−4.28) V and a perimidine–perimidine dimer linked via a 1,3′ bond is characterized by the lowest LUMO orbital. The optimization of the π–π stacking states was carried out in case of two polarized **1** molecules, where the stability of the dicationic state **π-[1]_2_^2+^** obtained by coupling of two radical cations **(1)^•+^** with an intermolecular distance of 3.68 and 3.46 Å between the planes of the two perimidines and two carbazoles, respectively. The interaction of di(radical dication)s **(1)^2^^•+^** is weaker, which is probably the result of the repulsion of carbazole units and the distances between the perimidines slightly increase to 3.75 Å. On the other hand, the pairing of radical cations between carbazole segments within adjacent dimers cannot be ruled out, which ultimately contributes to the achievement of a non-spin state **π-[1]_2_^4+^** (Table S6).

### 3.2. Electrochemical Reduction and Oxidation of Monomers **1**–**3**

The first reduction peak is completely reversible in DCM solution for all compounds, where the maximum of the peak takes a similar value is at approximate value i.e., −1.73, −1.65 and −1.69 V for **1**, **2**, and **3**, respectively (See Supplementary Materials, Figure S1). Thus, it was established that the reaction between radical anions with spin density located on 14H-benz[4,5]isoquino[2,1-a]perimidin-14-one is not possible (Table S3).

**Figure 3:**
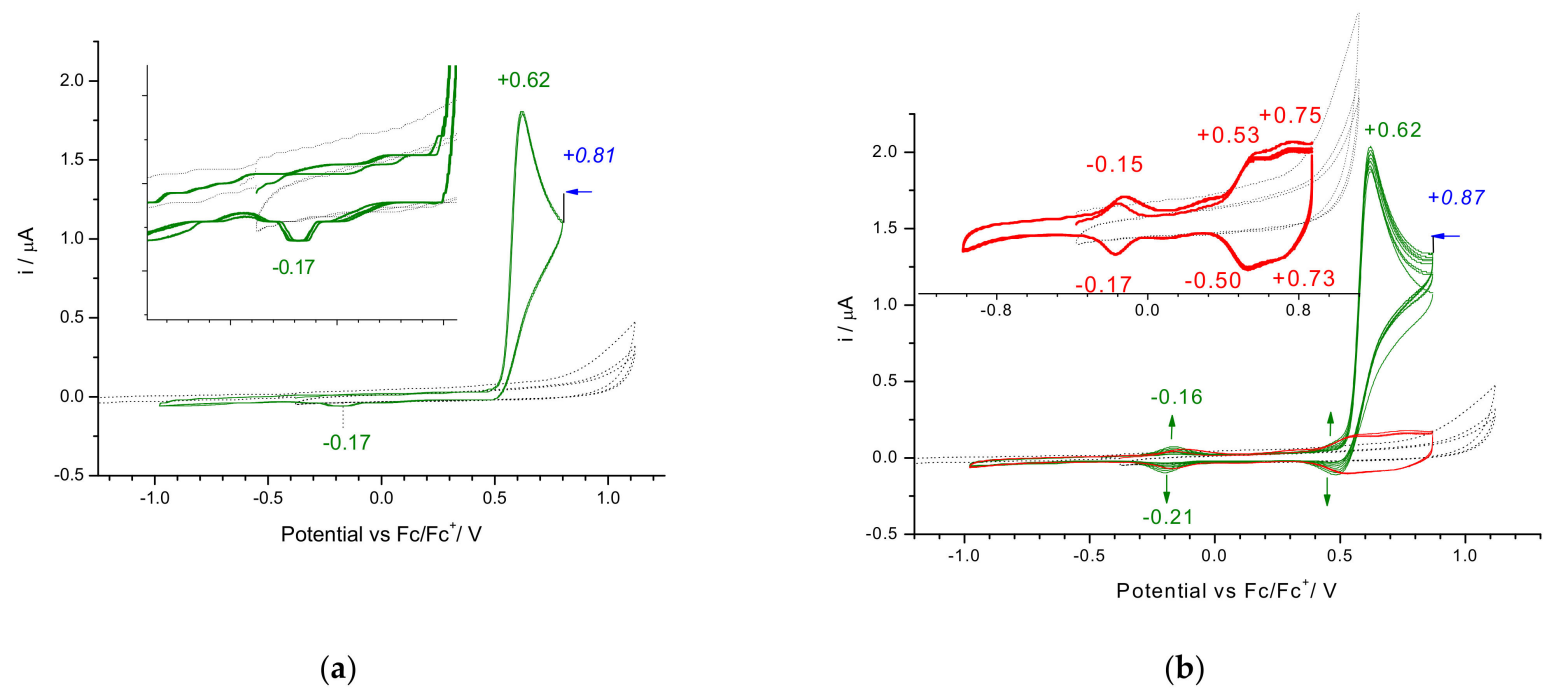

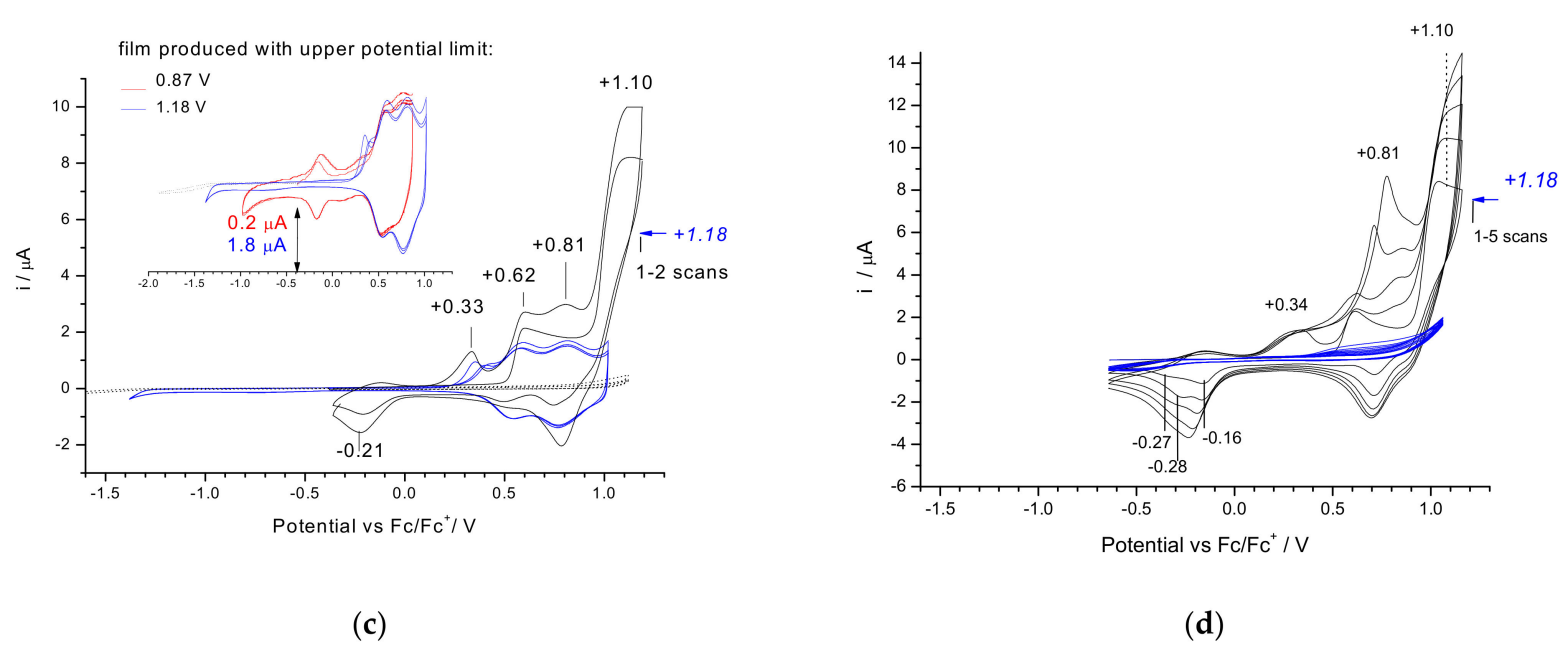
CVs limited to +0.81 V (**a**), +0.87 V (**b**) and +1.18 V (**c**,**d**) in 1 mM of **1** and 0.1 M Bu_4_NBF_4_ in DCM; potential sweep of **p(1)-I** (*red CVs*) (**b**,**c**), **p(1)-II** (*blue CVs*) (**c**) and **p(1)-III** (*blue CVs*) (**d**) in monomer-free in 0.1 M Bu_4_NBF_4_/DCM solution; 50 mV/s.

**Scheme 2:**
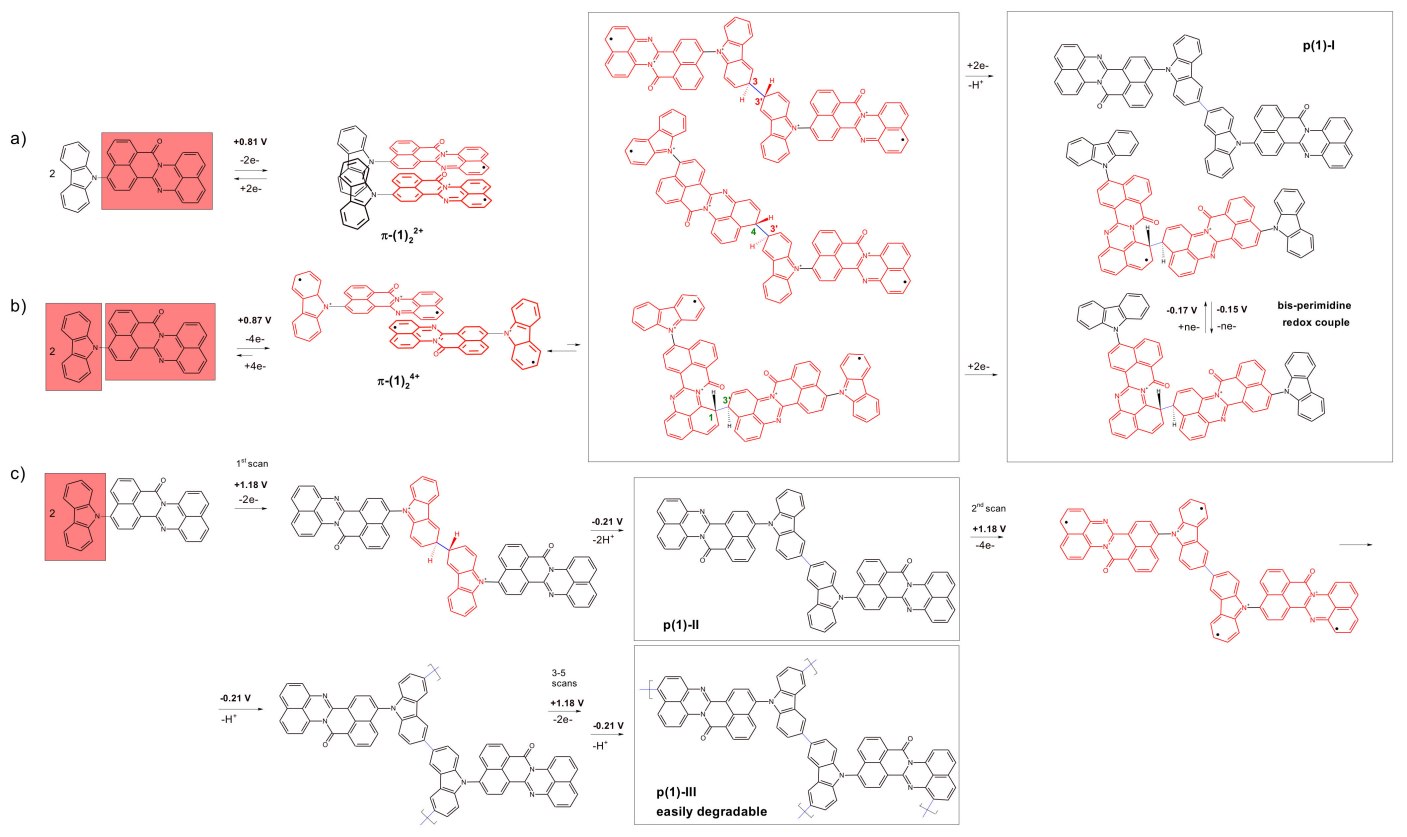

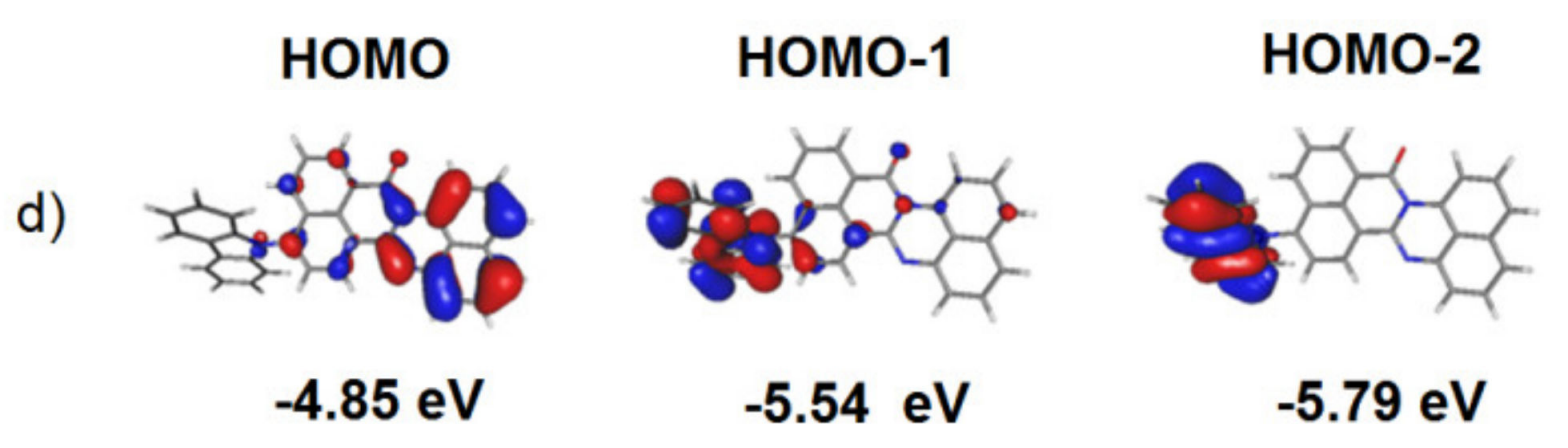
Proposed mechanism of **1** electrooxidation limited by the first (**a**), second (**b**) and third oxidation peak (**b**); with the production of **π-[1]_2_^2+^**, **π-[1]_2_^4+^** dimers (**a,b**) and solid product **p(1)-I** (**b**); **p(1)-II** and **p(1)-III** (**c**); polarized segment of **1** on a *red background*, new bonds in *blue*; shape of frontier orbitals of **1** (**d**).

Next, the upper range of polarization was limited after each oxidation peak and the reactivity of generated species of subsequent oxidation states was studied. The calculations show that the first and second oxidation states are separated by a small energy barrier, so it seems that the restriction after the first oxidation peak will cause the generation of diradical dication immediately. However, we tried to get radical cationic and diradical dicationic states of **1** compound by gradually limiting the upper value of the potential. The first oxidation peak at +0.62 is irreversible while limiting the upper value to +0.81 V, a slight reduction peak in the return half-cycle is observed (Figure 3a and Scheme 2a).

Already, a little shift of the upper potential to +0.87 V, still within the first oxidation peak, resulted in the deposition of the product **p(1)-I** with three redox couples—the first at potential −0.15/−0.17 V, the second and third in the form of wider current waves with oxidation peaks at +0.53, +0.75 and after polarity reversal at +0.70 and +0.50 V as reduction processes (Figure 3b). The potential of the first redox pair is characteristic for the redox activity of the bis-perimidine segment, which was reported in our previous work [61].

**Figure 4:**
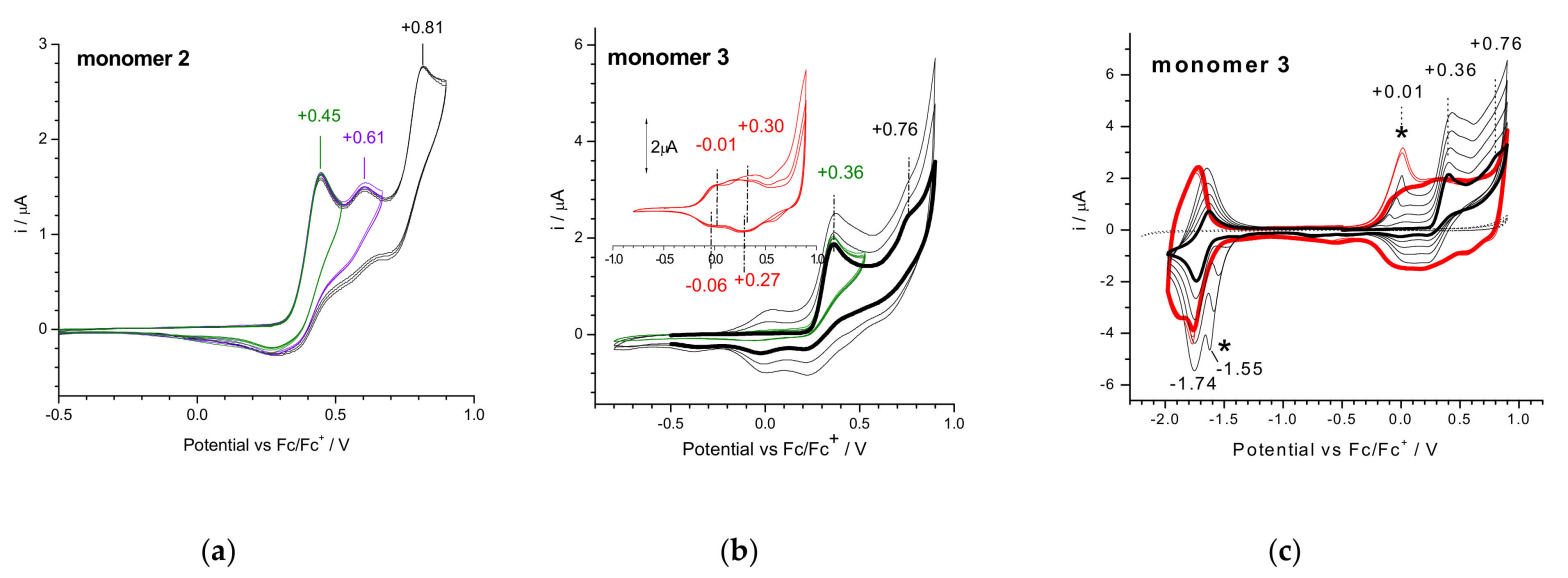
CVs limited after following oxidation peaks in 1 mM of **2** and 0.1 M Bu_4_NBF_4_/ DCM (**a**); CVs of electrodeposition after first (*green*) and second (*black*) oxidation peak (**a**,**b**), potential sweep between second oxidation and first reduction peaks (**c**) in 1 mM of **3** and 0.1 M Bu_4_NBF_4_/DCM and polarization of solid **p(3)** (*red*) in monomer-free 0.1 M Bu_4_NBF_4_/DCM solution (**b**,**c**); first CV cycle (bold); charge-trapping peaks (*); 50 mV/s.

The low potential of this redox system is characteristic for the protonated form of this adduct, which is undergoing a transition between states of dication and radical cation as this σ-dimer at −0.15/−0.17 V (Scheme 2b). This is consistent with our DFT results, where the LUMO level of the dication state of protonated bis-perimidine dimers is shifted relative to the HOMO level of the monomer by 1 eV, giving a potential of mentioned redox couple equal to approximately −0.2 V (Figure 2, Table S4). Even carrying out an analogous experiment in DMSO did not result in deprotonation of bis-perimidine dimers (Figure S2). The presence of two reversible redox states at −0.12/−0.17 V and +0.19/+0.10 V may indicate a transition between the dication and its corresponding radical cationic state of different σ-dimers linked through various positions of bis-perimidine and/or carbazole-perimidine (Figure 4, Table S4). On the other hand, the broad current wave recorded above +0.45 V also indicates the reversible oxidation of the product characterized by the greater π-electron delocalization, as if it could be in the case of deprotonated bis-carbazole junctions (Figure 3b, *red* CVs) [62,63]. It seems that the product of **(1)^2•+^** coupling identified as **p(1)-I** is a mixture of different dimers linked via the position of perimidine and carbazole; however, the efficiency of the subsequent reactions between oxidized dimers is low. DFT studies have also shown that the orbitals of HOMO and HOMO-1 dimers binding via carbazole segments are separated by a slight energy barrier, and the location of the HOMO-1 orbital is mainly located on perimidine units. Therefore, it was reasonable to determine whether we can also polarize the terminal (perimidine) units of this dimer in the applied potential window. Although the coupling of **(1)^2^**^•**+**^ through carbazoles to dimers was observed, the following coupling between bis-carbazole radical cationic and radical cationic of perimidine segments is already ineffective and two-dimensional bonding is inhibited by postulated π–π stacking interaction between aromatic rings (Scheme 2a,b). In conclusion, both the reactivity between **(1)^•+^** and **(1)^2•+^** species is low, which has been tried to explain further, based on spectroelectrochemical measurements.

In turn, stopping upper polarization at +1.18 V results in the intensive deposition of the product marked as **p(1)-II**, where triradical trication **(1)^3•+^** is formed within the potential range encompassing the next oxidation peak of the monomer. The spin density of **(1)^3•+^** is strongly centralized on carbazole and the coupling process should be running through this segment (Figure 3c and Scheme 2c). However, not only the higher oxidation state of the monomer is achieved in the range of this second oxidation peak, but also oxidation of the deposited product, as demonstrated by the higher current of the second oxidation peak relative to the first at +0.62 V. We observe an increase in currents above +0.3 V during the **p(1)-II** deposition, i.e., in a similar potential range as for **p(1)-I**; however, here no characteristic redox −0.15/−0.17 V pair, which we were observing previously. Instead, we discovered an irreversible reduction peak at −0.21 V from deprotonation of the intermediate products. However, this product slowly loses good adhesion, it dissolves or degrades during following CV cycles (Figure 3d). This may be related to the occurrence of subsequent processes related to the involvement of terminal perimidine units in the process of crosslinking of the **p(1)-II** to **p(1)-III** simultaneously decomposing under the applied oxidation. It also seems that π-conductivity is most influenced by the formation of a rigid π-conjugated backbone, i.e., through carbazole units, while competitive couplings with engagement both perimidine and carbazole segments do not result in a well-ordered conjugated structure, while the potential implemented for the oxidation of carbazole, unfortunately, causes a parallel degradation of bonds involving perimidine.

The presented experiment carried out with stepwise limitation of the upper oxidation potential of **1** shows that it is possible to control the polarity of a particular monomer segment and thus obtain the products significantly different in electrochemical properties.

Oxidation of **2** reveals three following anodic peaks with a maximum at +0.45, +0.61 and +0.81 V, all irreversible and each of these upper potential limits do not reveal new cathode or anode peaks in subsequent cycles (Figure 4a). The intensity of the second peak is lower than of the others and may rather indicate the oxidation of product obtained in the range of the first oxidation peak of the monomer. Arrangement of the HOMO and HOMO-1 orbitals of 2 occur on both thiophene rings (Table S2), therefore probably, protonated dimer bonding via thiophenes can be oxidized at this higher +0.61 V potential, because of π-conjugation breaking in this structure. Additionally, the stability of such adduct and its inability to deprotonate may result from the steric hindrance of the alkyl substituents. Polarization after the peak with the maximum at +0.81 V also did not provide the occurrence of effective polymerization, while calculations have shown that the HOMO-2 of 2 shifted relative to from its HOMO by 0.54 V and the orbital is already located mainly on perimidine.

**Scheme 3:**
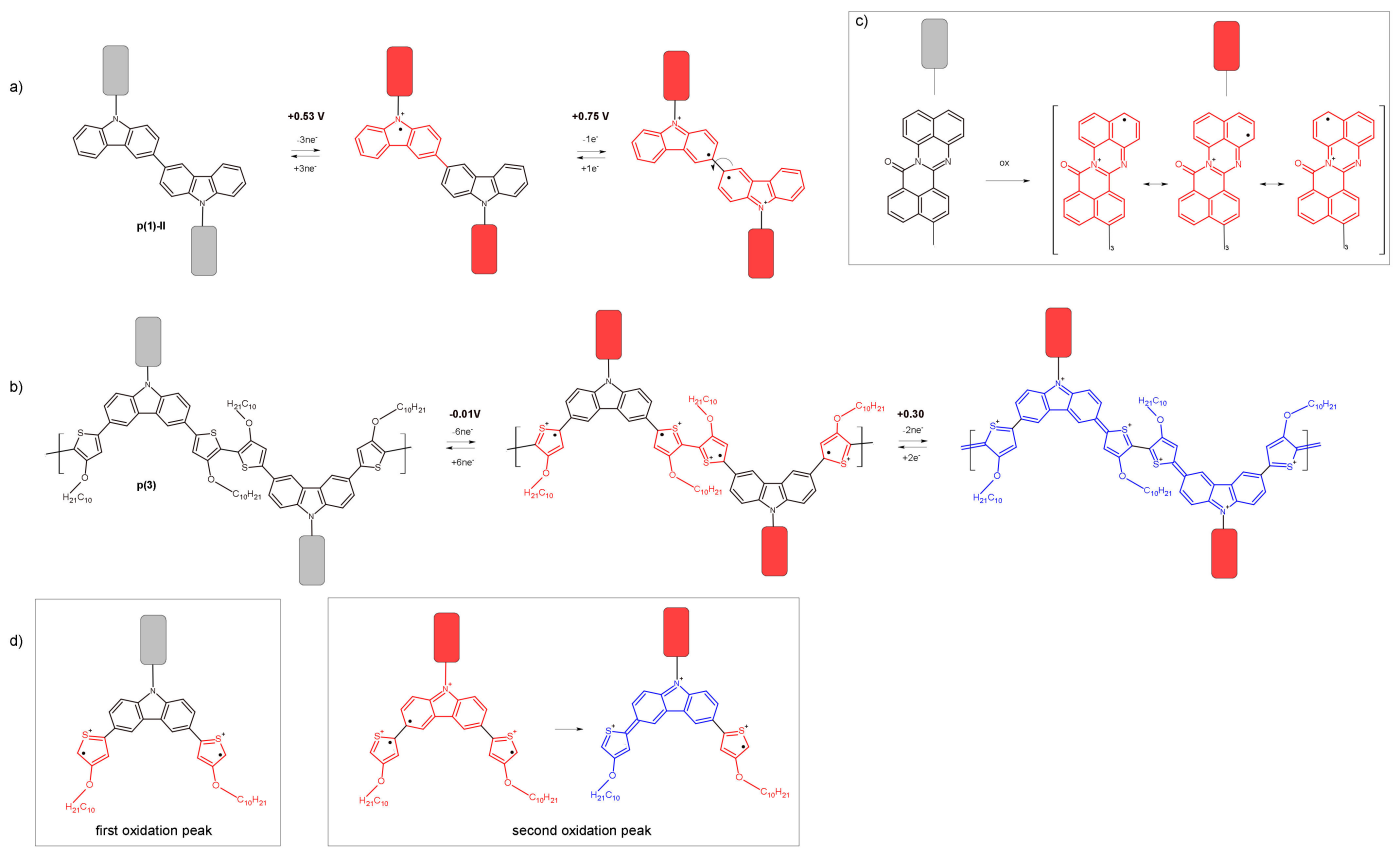
Electrooxidation of one-dimensional π-conjugated **p(1)-II** (**a**) and **p(3)** (**b**); radical cation (polaronic) state (*red*); dication (bipolaronic) state (*blue*). Perimidine side-group marked as a rectangle shape: neutral (*grey*) and oxidized moiety (*red*) (**c**); transformation of **3** under the influence of potential at first and second oxidation peak (**d**).

Compound **3** is characterized by the lowest first oxidation potential with a maximum at +0.36 V caused by the alkoxy group attached to thiophenes in their 4 and 4′ positions, which increases the spin density at unsubstituted positions through which the polymerization process can potentially take place. The first oxidation peak is irreversible and potential sweep limited after this one yields the diradical dication generation of **3** with the spin density located on both thiophene rings, on 3, 5 and 3′, 5′, despite this, did not observe any new peaks in subsequent scans (Figure 4b, *green CVs*). Applying 0.9 V potential as the upper limit with a second oxidation peak at +0.76 V results in just deposition of a conductive product with a very low oxidation onset at approx. −0.20 V and two reversible redox states at −0.01/−0.06 V and +0.30/+0.27 V with a wide range of potential (Figure 4b, *black CVs*). It seems that achieving the next oxidation state, with the strong intensified spin density on thiophenes, carbazole and perimidine (Table S2), as well as a much higher concentration of these species, resulted only in effective polymerization to a linear π-conjugated product. It should also be noted that the π-conjugated polymers could be formed only after the previous deprotonation of intermediate product—σ-dimer. When a higher oxidation state is achieved, it is also possible to oxidize both thiophenes and carbazole ring, and the co-planarity of these units allows for the recombination of radical cations of one thiophene and carbazole to the dication, then only one terminal thiophene group being in a reactive radical cationic form, which leads to the σ-dimer formation and its easier deprotonation (Scheme 3d). Reaching the second oxidation and first reduction peak of **3** results in an increase of currents in the range of these oxidation and reduction processes related to deposition of the electroconductive product **p(3)** being π-conjugated along thiophene-carbazole-thiophene segments. We observed total reversibility of **p(3)** electrooxidation in monomer-free electrolyte solution (Figure 4b, inset). Full cycle of alternating oxidation and reduction and both these opposite doping processes are also reversible as in ambipolar π-conjugated polymers. However, charge trapping effects were also observed, which was confirmed by the presence of sharp peaks preceding both anodic and cathodic doping (Figure 4c, charge release peaks marked with an asterisk), when alternately trapping both anions and cations after electrochemically oxidation and reduction occur, respectively. Particularly in low-porosity materials, the reversion of the polarity of polymer after oxidation or reduction often not enough to produce of neutral state. Further oxidation of the polymer is required to remove the cations involved during electroreduction, and vice versa, the only reduction of the polymer releases anions trapped during oxidation. 

### 3.3. In Situ UV-Vis-NIR Study of Polarization of **1**–**3** Monomers, Products **p(1)** and **p(3)**

**Figure 5:**
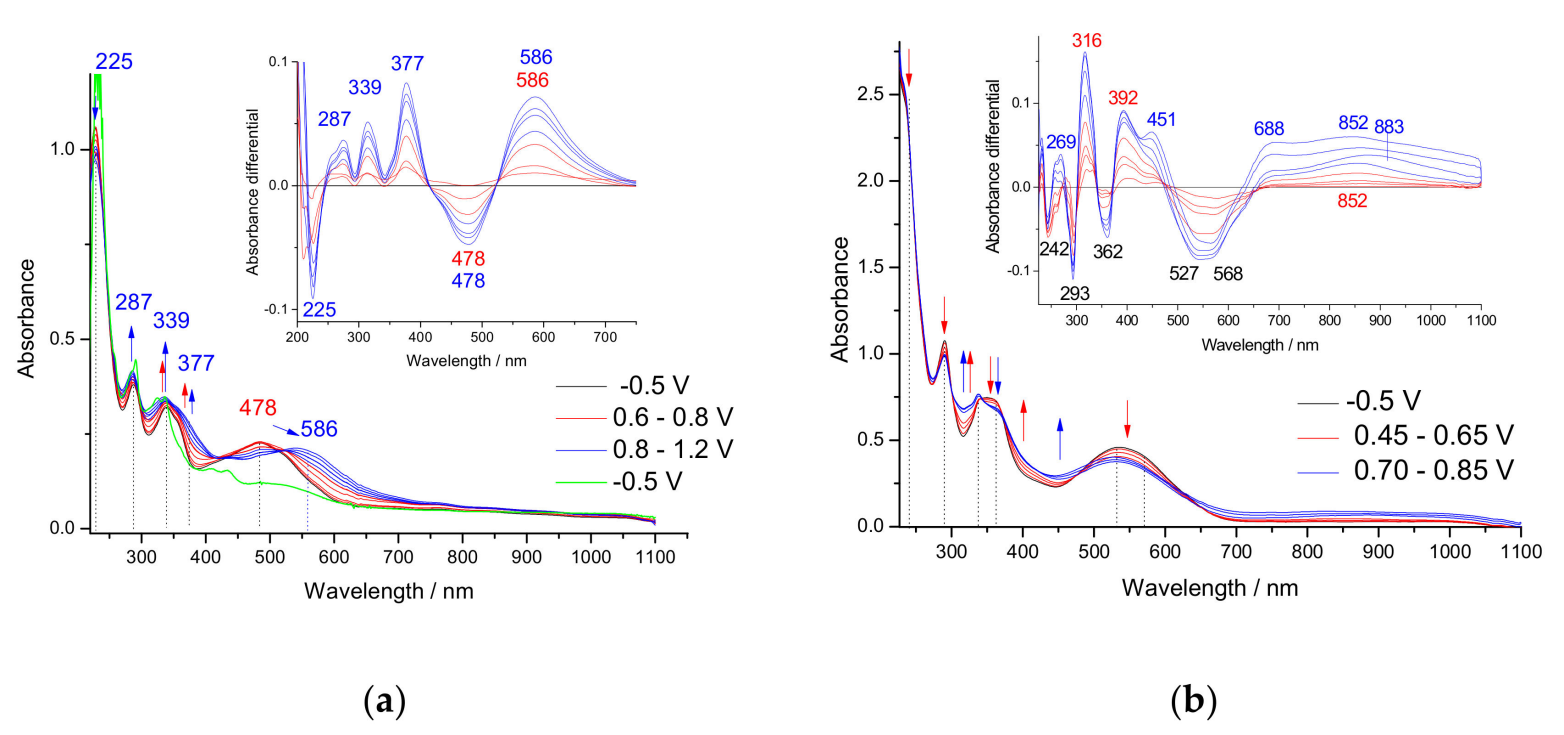
Change in absorbance of UV-Vis-NIR under polarization of diradical dication (*red*) and triradical trication production (*blue*) of **1** (**a**) and **2** (**b**) in 1mM solution of monomer in 0.1 M Bu_4_NBF_4_/DCM solution; difference absorbance vs wavelength (*inserts)*; neutral **p(1)-II** under -0.5 V polarization (*green*).

Gradual oxidation change in the range of the first oxidation peak of **1** (0.60–0.80 V) should involve the formation of a radical cation and then a diradical dication (Figure 5a, *red curves*). However, no characteristic bands from the radical cation of perimidine **(1)^•+^** (TD-DFT: 430, 534, 831 nm, Figure S3) or additionally from carbazole in the state of diradical dication **(1)^2•+^** (TD-DFT: 443, 726, 905 nm, Figure S3) were observed. Instead, a decrease was observed in bands from the neutral precursor (**1**) at 225 (n→π* transition from carbazole) and 478 nm (π→π* transition from perimidine), and an increase in the new ones at 287, 339, 377, and 586 nm. It seems that the increase and widening of the bands at 287, 339 nm may indicate an increase in π–π interactions between carbazole rings, and the band at 586 nm is characteristic of dicationic forms, which may be the result of the pairing of radical cationic forms. These spectral changes may indicate the occurrence of a disproportionation reaction, where from two diradical dication of precursor we can obtain one as a dication and one as neutral. However, due to the non-regeneration of the neutral precursor during oxidation and the reversibility of this process, the formation of π-dimers can be inferred, which may be a consequence of radical cations **(1)^•+^** pairing or diradical cations **(1)^2•+^** pairing, giving dicationic or tetracationic π-dimers: **π**-**(1)^2+^**, **π**-**(1)^4+^**, respectively. Therefore, the formation of the solid product **p(1)-I** is inefficient due to the equilibrium shift in this system towards π-dimers instead of σ-dimers (Scheme 2b).

Increasing the polarization potential to +1.0 V (Figure 5a, *blue curves*), corresponding to oxidation of carbazole segment, does not reveal the main bands characteristic for radical cationic or dicationic states of polycarbazoles backbone located in the range 770–870 nm [64], but only further the same increasing band at 586 nm, which was observed at the previous dicationic and tetracationic states in π-dimers (Figure 5a, *red curves*). Since the solid product **p(1)-II** successfully deposited in the previous potentiodynamic experiment in the sweep range between −0.35 and +1.18 V implementing two sweep cycles to limit degradation processes, it seems that the band at 586 nm should initially be assigned to a π-dimer, which can be transformed into a σ-dimer, with a parallel decrease in absorbance at 225 nm; however, it cannot be deprotonated at this high applied potential (+1.0 V). Only the return of the potential to −0.5 V allowed to deprotonation of dicationic σ-dimer to neutral dimer, for which the stepwise polarization process was checked in the next experiment. There was observed a broadening of π–π band and shift of its onset from 378 to 466 nm under in relation to the precursor band, which may indicate the formation under −0.5 V of a neutral dimer via carbazoles—**p(1)-II** (Figure S4). The absorbance of oxidized **p(1)-II** is characterized by an increase at 890 nm, which indicates the presence of radical cation states (Scheme 3a and Figure S4).

Oxidation of **2** was also performed in the range of the sequential oxidation peaks, where changes of the absorbance spectrum were studied. In the 0.45–0.65 V range, we observe the disappearance of the bands at 242, 293, 362, 527, and 568 nm, constantly present band at 527 nm and a slight increase in these new ones at 392 and 852 nm from the radical cation states (Figure 5b, *red curves*). In the range of the second oxidation peak (0.65–0.85 V), the absorption increases at 451 and 688 nm from dicationic state (recombination of radical cations of one thiophene and carbazole) with the simultaneous presence of the previously observed radical cation state at 852 nm (radical cation located on a perimidine), while the new one comes at 883 nm from the radical cation of the second thiophene (Figure 5b, blue curves). Similar behavior occurs during **3** oxidation, but we see a high efficiency in the occurrence of radical cationic state of the products, as indicated earlier, where σ-dimer can be deprotonated more easily in this case, as opposed to products of **2**.

**Figure 6:**
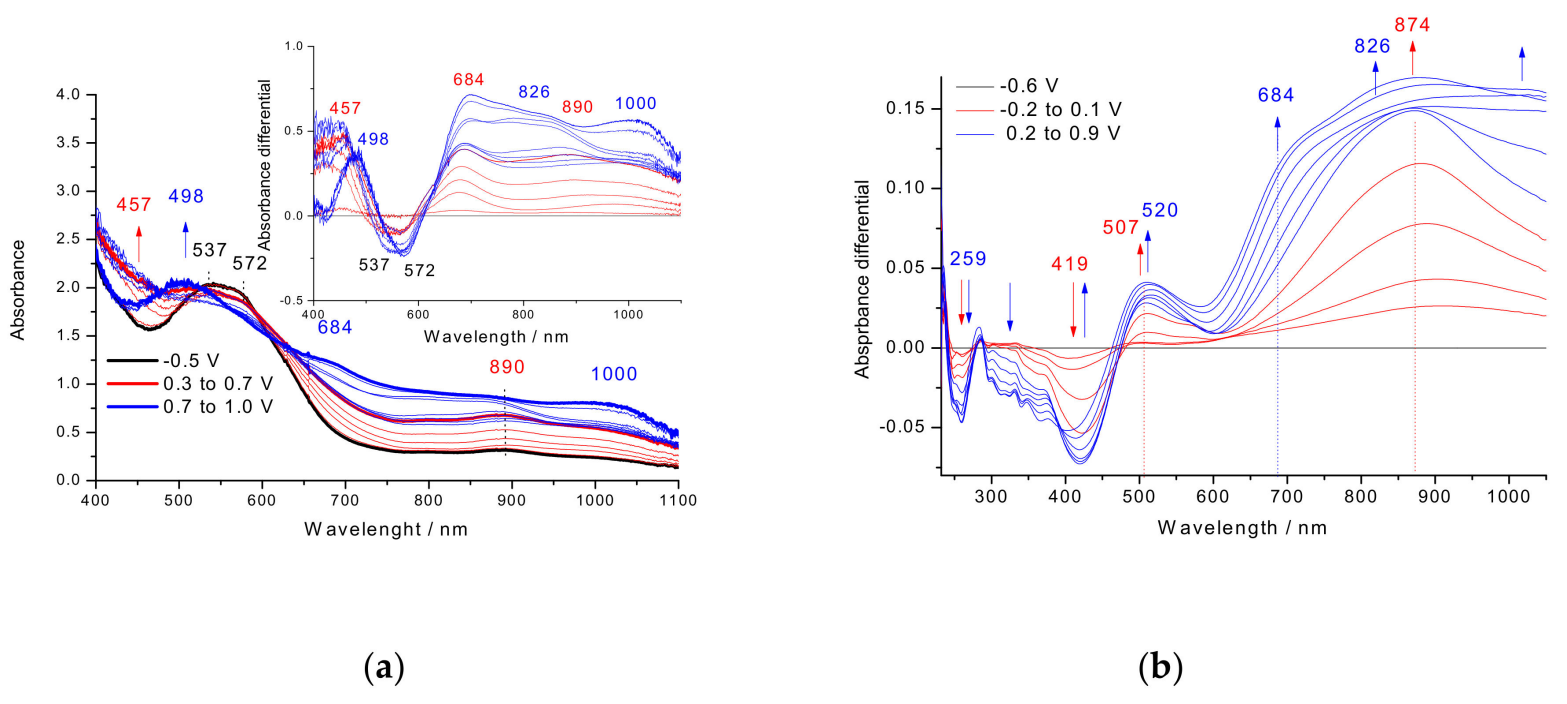
Change in absorbance of UV-Vis-NIR at the first (*red*) and second oxidation peak (*blue*) of **3** in 1mM solution of monomer in 0.1 M Bu_4_NBF_4_/DCM solution, difference absorbance vs wavelength (*insert*) (**a**), difference UV-Vis-NIR absorbance during oxidative polarization of **p(3)** in 0.1 M Bu_4_NBF_4_/DCM solution (**b**).

Changes during the electrooxidation of **3** are shown in Figure 6a. After reaching the potential of +0.3 V, an increase in absorbance at 684 and 826 nm was observed with a simultaneous decrease in absorbance at 537 and 572 nm (Figure 6a, *red curves*). The increase in absorbance in the range above 700 nm indicates the formation of a solid product, which is simultaneously oxidized with an increase of the band 1000 nm from bipolarons (Figure 6a, *blue curves*). Next, product **p(3)** was successfully deposited on the ITO electrode, and the absorbance changes (Figure S5) and differences in absorbance changes during polarization of this film are shown in Figure 6b. In the first stage of oxidation, a distinct band is formed at 507 and 874 nm, which most probably comes from the radical cation of thiophene in thiophene-carbazole-thiophene segments (Scheme 3b). Gradually increasing the potential to +0.9 V causes a decrease of the characteristic carbazole bands in the range 250–370 nm, which indicates the involvement of these units in the process of dication formation with absorption at 684 nm and due to extensive π-conjugation reaching values above 1000 nm. Bands at 520 and 826 nm are derived from the radical cation of the perimidine, which, as side moieties of the rigid π-conjugated thiophene-carbazole-thiophene backbone, retained only a characteristically isolated molecule, incapable of crosslinking or π–π-stacking. The complete reverse of potential reveals the gradual renewal of the initial spectrum, which indicates the stability of this π-conjugated product during its oxidative polarization (Figure 6b).

## 4. Conclusions

A search for new precursors of 2D and even 3D π-conjugated polymers and choice of polymerization techniques is important for development in the area of materials possessing high charge-carrier mobility. In contrast to the development of linear conjugated polymers, the successive increase of dimension by multiple covalently bonded giving two- and multi-directed π-conjugated polymers remains less explored. This is mostly because of the enormous synthetic challenges of monomers and restricting long-range ordered polymerization in 2D while keeping structural control at the molecular level. The combination of computational and electrochemical techniques made it possible to fine-tune the oxidation state of the precursor, which enabled the control of the polymerization process in the direction of 1D. However, two-dimensions propagation via dimers and between side groups in linear π-conjugated products was inhibited due to perimidine’s strong π-stacking tendency. Thus, this work also demonstrates the formation of peculiar species of perimidine radical cations and diradical dication, which can lead to a deeper fundamental understanding of the mechanism of dimer formation between different conjugated aromatic systems.

## Data Availability

All data presented in this study is available in M. Czichy, P. Janasik, P. Wagner.

## Supplementary Materials

The following are available online at https://www.mdpi.com/article/10.3390/ma14092167/s1. Figure S1: CV curves limited by first (green), second (red) and third oxidation (blue) and reduction peaks (black) registered in 1mM solution of 1 (a), 2 (b), 3 (c); in 0.1 M Bu4NBF4/DCM. p(1)-I deposited in the range −0.20–0.87 V while scanning between −1.89 and +0.87 V–(a) insert; 50 mV/s. Figure S2: CVs limited after first oxidation peaks to +0.89 V in 5 mM of 1 and 0.1 M Bu4NBF4/DMSO; product under polarization in monomer-free 0.1 M Bu4NBF4/DMSO (insert); 50 mV/s. Figure S3: Simulated UV-Vis-NIR spectra of **1** states, calculated at TDDFT/CAM-B3LYP/6-31G(d)/CPCM(DCM). Figure S4: UV-Vis-NIR spectrum for neutral monomer **1** (*black*); neutral **p(1)-II** under −0.5 V polarization (*green*); **p(1)-II** oxidized at 1V (*red*) in 0.1 M Bu_4_NBF_4_/DCM solution. Figure S5: Change in absorbance of UV-Vis under polarization of **p(3)** in the range of polaron (*red*) and bipolaron forming (*blue*) in 0.1 M Bu_4_NBF_4_/DCM solution. Table S1: Cartesian coordinates of the optimized structures of perimidine-carbazole monomers, calculated at B3LYP hybrid functional combined with 6-31G(d) basis set. Table S2: Shape of frontier orbitals of monomers calculated at B3LYP/6-31G(d)/CPCM(DCM). The isovalue is equal to 0.03 e^−^/au^3^ in each case. Table S3: Spin density of oxidized and reduced form monomers calculated at B3LYP/6-31G(d)/CPCM(DCM), isovalue is equal to 0.005 e^−^/au^3^. Table S4: Shape of frontier orbitals of selected dimers calculated at B3LYP/6-31G(d)/CPCM(DCM). The isovalue is equal to 0.03 e^−^/au^3^ in each case. Table S5: Summary of electrochemical parameters of monomers in 0.1 M Bu_4_NBF_4_/ DCM, potentials vs Fc|Fc^+^. Table S6: Optimized structures of **1** π-dimers of radical cations (left) and di(radical cations) (right), calculated at ϖB97X-D functional combined with 6-31G(d,p) basis set.
