# Supplementary material for: Electrochemical and Spectroelectrochemical Studies on the Reactivity of Perimidine–Carbazole–Thiophene Monomers towards the Formation of Multidimensional Macromolecules versus Stable π-Dimeric States
Source: Materials (Basel). 2021 Apr 23;14(9):2167. doi: 10.3390/ma14092167 (PMC8122979; doi:10.3390/ma14092167)
Supplement: Supplementary file 1 [file materials-14-02167-s001.zip › materials-1188300 - SM.pdf]

Electrochemical and Spectroelectrochemical Studies on the Reactivity of Perimidine–Carbazole–Thiophene Monomers Towards the Formation of Multidimensional Macromolecules versus Stable π-Dimeric States

Malgorzata Czichy [1,*], Patryk Janasik [1], Pawel Wagner [2], David L. Officer [2] and Mieczyslaw Lapkowski [1,3]

[1] Faculty of Chemistry, Silesian University of Technology, M. Strzody 9, 44-100 Gliwice, Poland; Patryk.Janasik@polsl.pl (P.J.); Mieczyslaw.Lapkowski@polsl.pl (M.L.)
[2] ARC Centre of Excellence for Electromaterials Science and the Intelligent Polymer Research Institute, University of Wollongong, Wollongong, NSW 2519, Australia; pawel@uow.edu.au (P.W.); davido@uow.edu.au (D.L.O.)
[3] Centre of Polymer and Carbon Materials, Polish Academy of Sciences, 34 Curie-Sklodowska Str., 41-819 Zabrze, Poland
* Correspondence: Malgorzata.Czichy@polsl.pl

Table S1. Cartesian coordinates of the optimized structures of perimidine-carbazole monomers, calculated at B3LYP hybrid functional combined with 6-31G(d) basis set.

1				2				3			
C	-2.30476	5.08792	-2.55706	C	-2.90784	8.49579	-4.83343	C	-2.94320	8.46374	-4.80669
C	-1.22811	5.84586	-2.12260	C	-2.16812	9.45530	-4.15926	C	-2.20761	9.42666	-4.13279
C	-0.13650	5.21946	-1.52690	C	-1.24375	9.06432	-3.19408	C	-1.27743	9.03967	-3.17161
C	-2.31261	3.69896	-2.40660	C	-2.74028	7.13614	-4.55926	C	-2.76550	7.10457	-4.53639
C	-1.22147	2.99819	-1.80319	C	-1.80694	6.67235	-3.57967	C	-1.82575	6.64479	-3.56111
C	0.95775	5.97998	-1.08416	C	-0.49091	10.02995	-2.50666	C	-0.52851	10.00871	-2.48466
C	2.05142	5.35169	-0.48731	C	0.43513	9.63757	-1.53942	C	0.40347	9.62028	-1.52152
C	2.05915	3.96571	-0.32947	C	0.61435	8.28400	-1.25368	C	0.59246	8.26730	-1.23938
C	0.97119	3.20322	-0.76895	C	-0.13344	7.31770	-1.93578	C	-0.15134	7.29766	-1.92111
C	-0.03140	1.06691	-1.01364	C	-0.64494	5.02564	-2.27286	C	-0.64764	5.00295	-2.26282
C	-2.17265	0.71123	-2.01434	C	-2.25776	4.24321	-3.85392	C	-2.25876	4.21297	-3.84170
C	-2.05564	-0.68015	-1.80301	C	-1.99625	2.90680	-3.48014	C	-1.98414	2.87724	-3.47438
C	0.10967	-0.32172	-0.78841	C	-0.36549	3.69684	-1.87904	C	-0.35603	3.67513	-1.87479
C	-0.91022	-1.20160	-1.17808	C	-1.04968	2.63016	-2.47922	C	-1.03196	2.60466	-2.47763
C	-3.07269	-1.56530	-2.21710	C	-2.66890	1.83418	-4.10183	C	-2.64859	1.80132	-4.09906
C	-2.94291	-2.93616	-2.02488	C	-2.39407	0.51947	-3.74250	C	-2.35945	0.48754	-3.74760
C	-1.81097	-3.45478	-1.40527	C	-1.45965	0.24301	-2.75026	C	-1.41869	0.21503	-2.76020
C	-0.78319	-2.60494	-0.95143	C	-0.78383	1.28475	-2.08491	C	-0.75137	1.25973	-2.09099
C	0.38418	-3.11029	-0.31512	C	0.17532	1.02826	-1.06665	C	0.21412	1.00781	-1.07723
C	1.39781	-2.21313	0.04338	C	0.86270	2.10888	-0.50069	C	0.89109	2.09319	-0.50744
C	1.26612	-0.84776	-0.18191	C	0.59832	3.41652	-0.89170	C	0.61271	3.39975	-0.89125
N	-1.16295	1.60437	-1.62038	N	-1.58802	5.32148	-3.25297	N	-1.59586	5.29459	-3.23908
N	0.98634	1.87070	-0.61299	N	0.04413	6.01796	-1.65411	N	0.03605	5.99853	-1.64333
N	0.57282	-4.51305	-0.00828	N	0.46631	-0.30163	-0.57299	N	0.52488	-0.32144	-0.59300
O	-3.18762	1.10162	-2.55758	O	-3.08879	4.41850	-4.72362	O	-3.09399	4.38459	-4.70808
C	-0.26521	-5.34529	0.67237	C	-0.41173	-1.23348	-0.10614	C	-0.33698	-1.27177	-0.13261
C	0.28142	-6.61003	0.78368	C	0.25142	-2.37178	0.30330	C	0.34618	-2.40156	0.26805
C	1.50693	-6.56344	0.16400	C	1.59093	-2.13982	0.09886	C	1.68136	-2.14517	0.06531
C	1.66471	-5.27162	-0.30050	C	1.69483	-0.86821	-0.42512	C	1.76305	-0.86852	-0.44992
C	2.82397	-4.89889	-0.99353	C	2.94429	-0.33237	-0.75198	C	3.00326	-0.30949	-0.77386
C	3.82274	-5.86942	-1.18961	C	4.08829	-1.11597	-0.52051	C	4.16058	-1.07429	-0.54712
C	3.65382	-7.18267	-0.70294	C	3.98371	-2.42157	0.02741	C	4.07888	-2.38466	-0.00711
C	2.48230	-7.54303	-0.01671	C	2.70601	-2.93936	0.34027	C	2.81032	-2.92687	0.30162
C	-0.37585	-7.65047	1.43874	C	-0.41667	-3.47774	0.82549	C	-0.30191	-3.52309	0.78183
C	-1.63004	-7.37757	2.00904	C	-1.82316	-3.41495	0.94853	C	-1.70912	-3.48657	0.90536
C	-2.18940	-6.08576	1.92216	C	-2.50296	-2.23493	0.54773	C	-2.40999	-2.31557	0.51442
C	-1.50637	-5.05333	1.25536	C	-1.80144	-1.13629	0.02374	C	-1.72821	-1.20021	-0.00085
H	-3.14984	5.58349	-3.01905	H	-3.62498	8.80887	-5.58236	H	-3.66490	8.77378	-5.55248
H	-1.25010	6.92156	-2.25270	H	-2.31977	10.50261	-4.39309	H	-2.36695	10.47347	-4.36371
H	-3.20650	3.25210	-2.78676	H	-3.37772	6.51795	-5.15485	H	-3.40064	6.48337	-5.13133
H	0.96697	7.05745	-1.19952	H	-0.61857	11.08533	-2.71696	H	-0.66368	11.06369	-2.69224
H	2.89392	5.93967	-0.14670	H	1.01437	10.38344	-1.01056	H	0.97981	10.36872	-0.99312
H	2.91164	3.48511	0.13486	H	1.33483	7.98709	-0.50130	H	1.31760	7.97342	-0.49029
C	-0.13337	3.80964	-1.36863	C	-1.06511	7.68651	-2.90709	C	-1.08869	7.66242	-2.88841

H	-3.96976	-1.20368	-2.70476
H	-3.72523	-3.60365	-2.36331
H	-1.75342	-4.52693	-1.30108
H	2.08068	-0.20701	0.13187
H	2.29499	-2.57271	0.53125
H	-1.94008	-4.06439	1.20653
H	-3.15141	-5.88619	2.37596
H	-2.16629	-8.16174	2.52739
H	0.06516	-8.63584	1.51459
H	2.96185	-3.89911	-1.38035
H	4.72816	-5.60695	-1.72121
H	4.43099	-7.91886	-0.86180
H	2.34571	-8.54945	0.35699

H	-3.40329	2.00502	-4.87953
H	-2.90741	-0.29332	-4.24045
H	-1.27088	-0.79520	-2.52565
H	1.15381	4.20773	-0.40391
H	1.59574	1.93964	0.27795
H	-2.33828	-0.24295	-0.26302
H	-3.57872	-2.15997	0.64306
H	0.14201	-4.35201	1.13227
H	3.04648	0.65550	-1.17869
H	5.05408	-0.69875	-0.77305
H	2.58340	-3.93063	0.75762
C	-2.58163	-4.56163	1.50130
C	5.19676	-3.23823	0.26909
C	-3.95825	-4.61251	1.67954
C	-4.39862	-5.83090	2.22675
C	-3.38075	-6.74612	2.48299
S	-1.93345	-6.02432	2.02325
C	5.21691	-4.52238	0.80002
C	6.51154	-5.04966	0.91767
C	7.52377	-4.19193	0.48286
S	6.78461	-2.77786	-0.04578
C	-5.83762	-6.15939	2.52681
C	6.74132	-6.43073	1.47343
C	8.21527	-6.83398	1.52710
H	6.19668	-7.16438	0.84088
H	6.32546	-6.47384	2.50311
H	8.65487	-6.82922	0.50683
H	8.30499	-7.85847	1.94592
H	8.78384	-6.13518	2.17723
C	-6.80855	-5.03007	2.18007
H	-5.93289	-6.38700	3.61040
H	-6.12536	-7.06355	1.94830
H	-6.56490	-4.11749	2.76504
H	-7.84509	-5.34213	2.42737
H	-6.75868	-4.79665	1.09502

H	-3.38709	1.96893	-4.87357
H	-2.86634	-0.32765	-4.24825
H	-1.21861	-0.82242	-2.54251
H	1.16200	4.19396	-0.40130
H	1.62828	1.92829	0.26816
H	-2.28106	-0.31429	-0.28008
H	-3.48693	-2.26085	0.61052
H	0.27239	-4.38972	1.08131
H	3.08916	0.68245	-1.19449
H	5.11887	-0.63841	-0.79667
H	2.70514	-3.92269	0.71284
C	-2.44625	-4.65202	1.44768
C	5.30664	-3.17992	0.23174
C	-3.82178	-4.73091	1.62404
C	-4.23320	-5.96585	2.15613
C	-3.20280	-6.86352	2.40989
S	-1.77039	-6.10769	1.95739
C	5.35107	-4.46579	0.75695
C	6.65328	-4.96919	0.87716
C	7.64754	-4.08980	0.44896
S	6.88543	-2.68722	-0.07643
O	-5.52231	-6.28593	2.42847
C	-6.62775	-5.50095	2.15189
C	-6.88284	-5.39839	0.64874
H	-6.50422	-4.49102	2.60020
H	-7.51656	-5.97795	2.61612
O	6.85538	-6.21790	1.37788
C	8.00797	-6.96062	1.62144
C	9.34021	-6.28983	1.28552
H	7.93877	-7.90351	1.03895
H	8.02040	-7.22930	2.69888
H	-7.03067	-6.41230	0.22031
H	-6.03608	-4.90705	0.12843
H	-7.79855	-4.79681	0.46875
H	9.39907	-6.05827	0.20135
H	10.17071	-6.98533	1.52997
H	9.47747	-5.36936	1.89107

Table S2. Shape of frontier orbitals of monomers calculated at B3LYP/6-31G(d)/CPCM(DCM). The isovalue is equal to 0.03 e⁻/au^3 in each case.

	LUMO+1 / eV	LUMO / eV	HOMO / eV	HOMO-1 / eV	HOMO-2 / eV
**1**	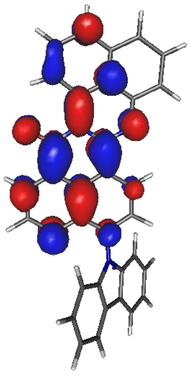<br>**-1.32**	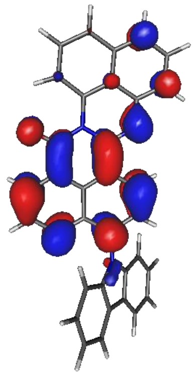<br>**-2.68**	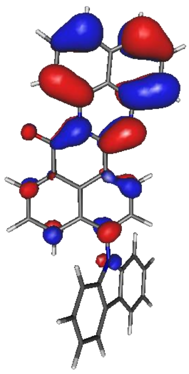<br>**-4.85**	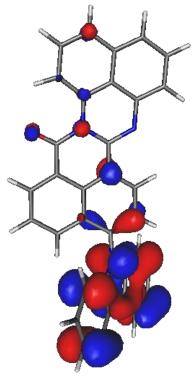<br>**-5.54**	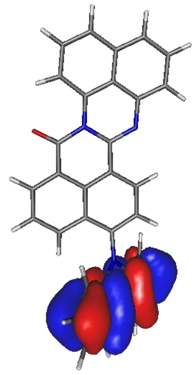<br>**-5.79**
**2**	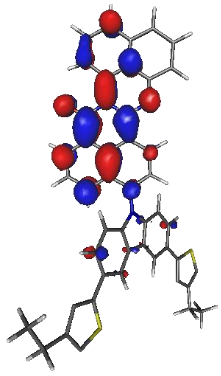<br>**-1.44**	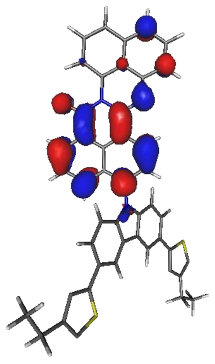<br>**-2.81**	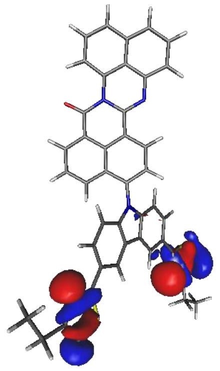<br>**-4.40**	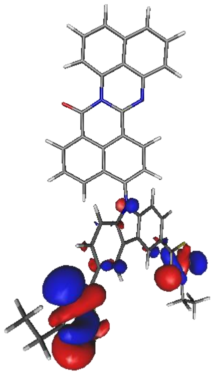<br>**-4.48**	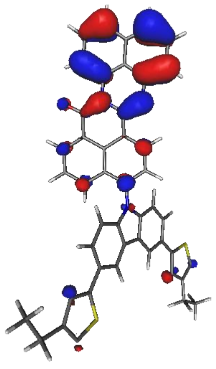<br>**-4.94**
**3**	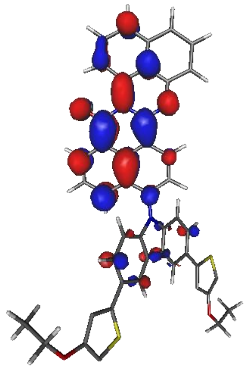<br>**-1.43**	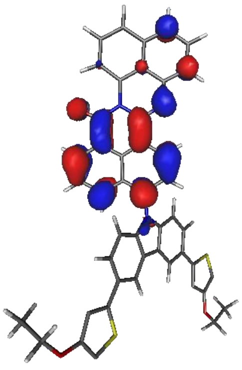<br>**-2.80**	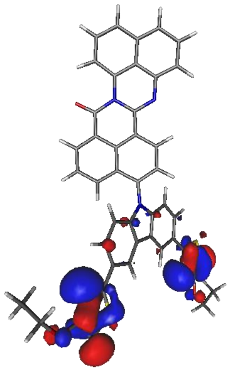<br>**-4.25**	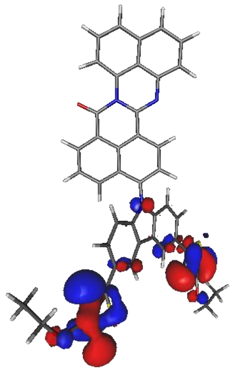<br>**-4.33**	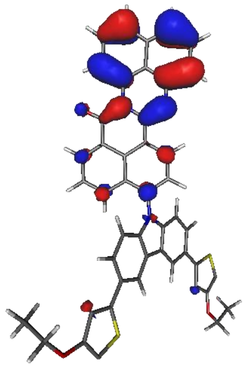<br>**-4.93**

Table S3. Spin density of oxidized and reduced form monomers calculated at B3LYP/6-31G(d)/CPCM(DCM), isovalue is equal to 0.005 e⁻/au³.

	**Diradical dianion** q=-2 m=3	**Radical anion** q=-1 m=2	**Radical cation** q=1 m=2	**Diradical dication** q=2 m=3	**Triradical trication** q=3 m=4
**1**	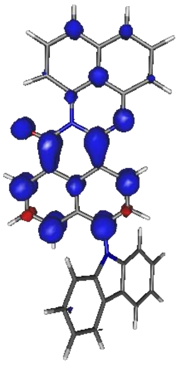	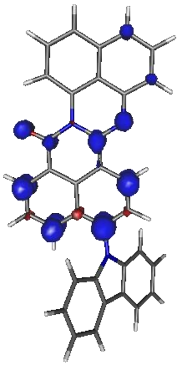	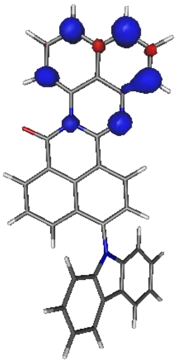	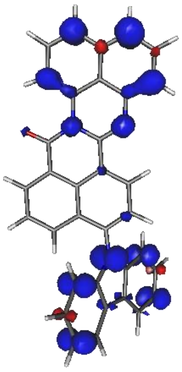	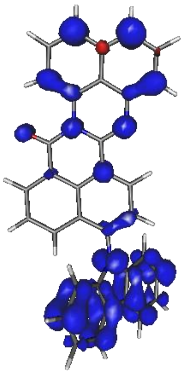
**2**	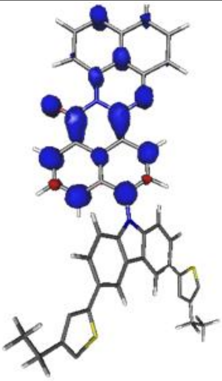	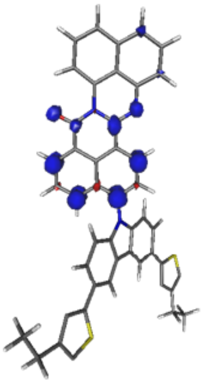	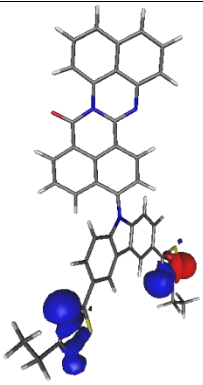	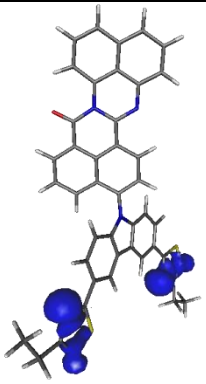	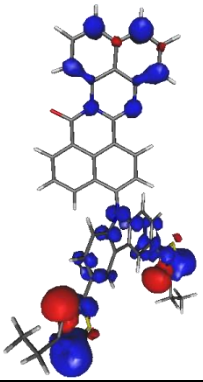
**3**	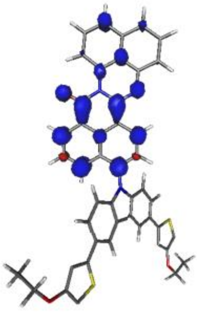	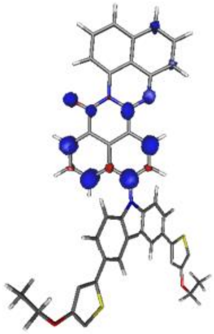	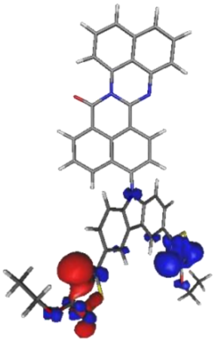	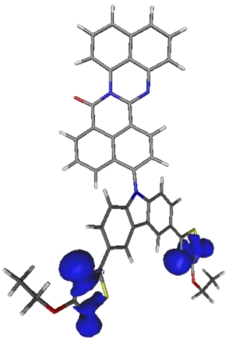	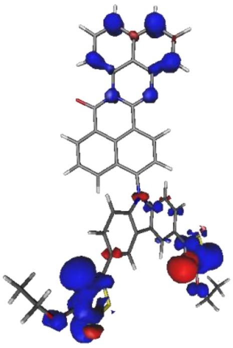

Table S4. Shape of frontier orbitals of selected dimers calculated at B3LYP/6-31G(d)/CPCM(DCM). The isovalue is equal to 0.03 e⁻/au^3 in each case.

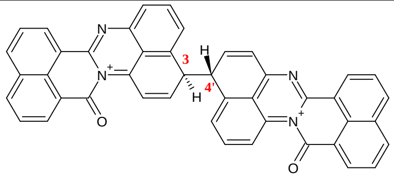

LUMO / eV	HOMO / eV
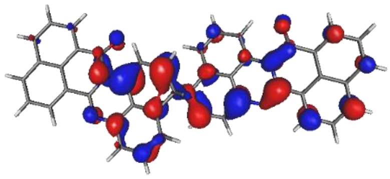	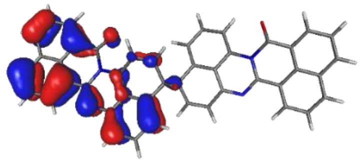
**-3.94**	**-6.51**

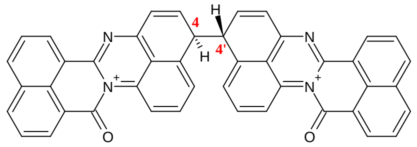

LUMO / eV	HOMO / eV
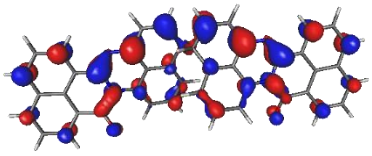	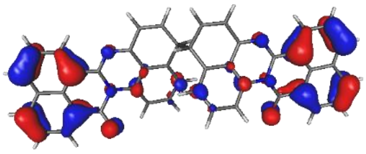
**-3.89**	**-6.80**

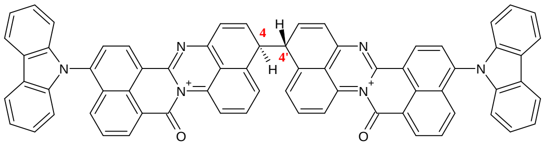

LUMO / eV	HOMO / eV	HOMO-1 / eV
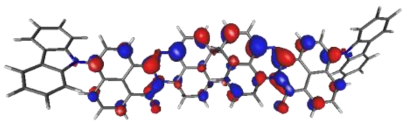	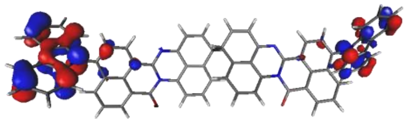	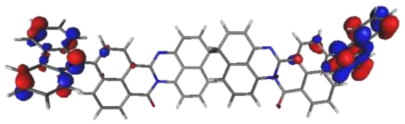
**-3.87**	**-5.73**	**-5.73**

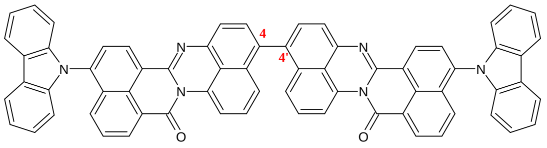

LUMO / eV	HOMO / eV	HOMO-1 / eV
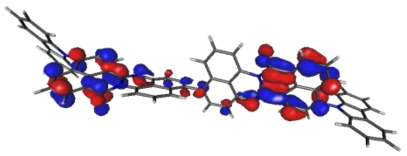	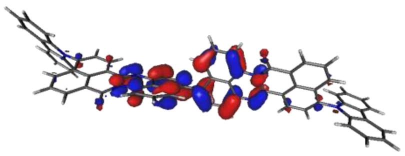	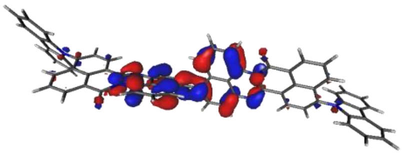
**-2.53**	**-5.08**	**-5.24**

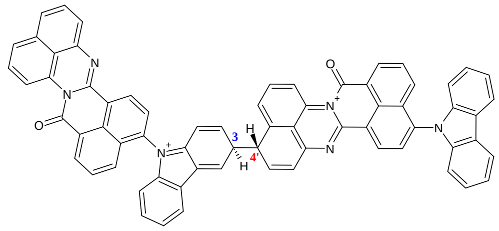

LUMO / eV	HOMO / eV	HOMO-1 / eV
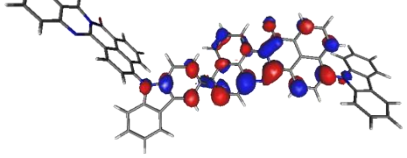	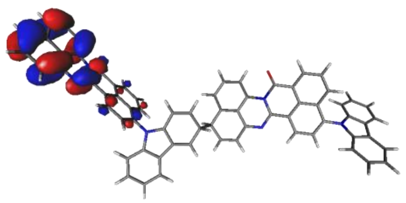	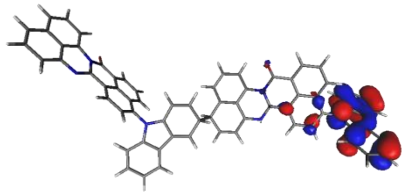
**-4.24**	**-5.54**	**-6.08**

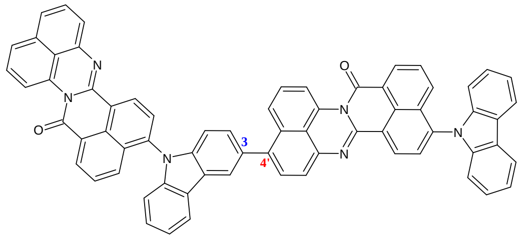

LUMO / eV	HOMO / eV	HOMO-1 / eV
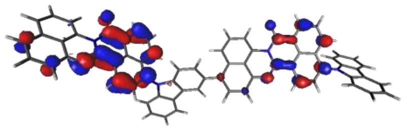	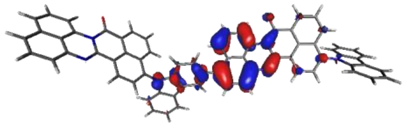	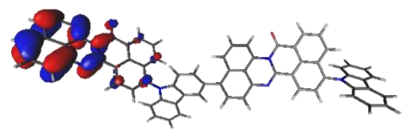
**-2.50**	**-5.01**	**-5.17**

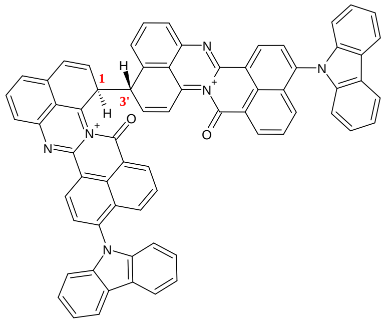

LUMO / eV	HOMO / eV	HOMO-1 / eV
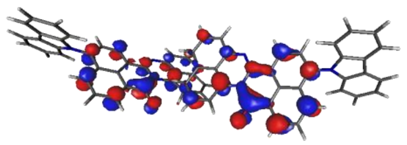	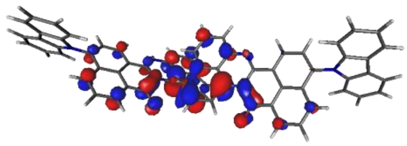	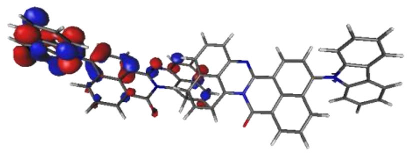
**-4.28**	**-5.94**	**-5.99**

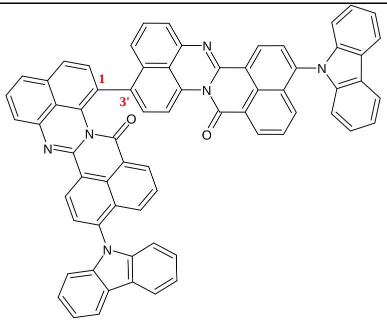

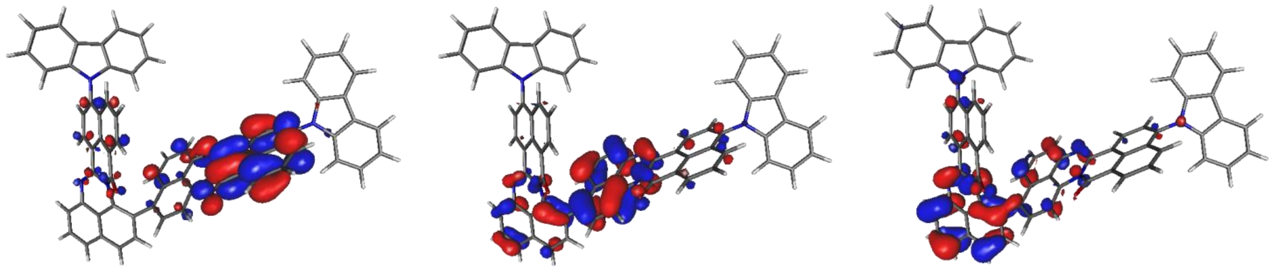

LUMO / eV	HOMO / eV	HOMO-1 / eV
**-2.50**	**-5.05**	**-5.28**

Table S5. Summary of electrochemical parameters of monomers in 0.1 M Bu_4NBF_4/ DCM, potentials vs Fc|Fc$^+$.

<table>
<tr><th>Abbreviation</th><th>E_{ox1}/ V</th><th>E_{ox2} / V</th><th>E_{ox3} / V</th><th>E_{red1} / V</th><th>HOMO $_{electr.}$ / eV</th><th>LUMO $_{electr.}$/eV</th></tr>
<tr><td>1</td><td colspan="2">+0.62</td><td>+1.10</td><td>-1.73</td><td>-5.61</td><td>-3.48</td></tr>
<tr><td>2</td><td>+0.45</td><td>+0.61</td><td>+0.81</td><td>-1.65</td><td>-5.45</td><td>-3.57</td></tr>
<tr><td>3</td><td colspan="2">+0.36</td><td>+0.76</td><td>-1.69</td><td>-5.34</td><td>-3.53</td></tr>
</table>

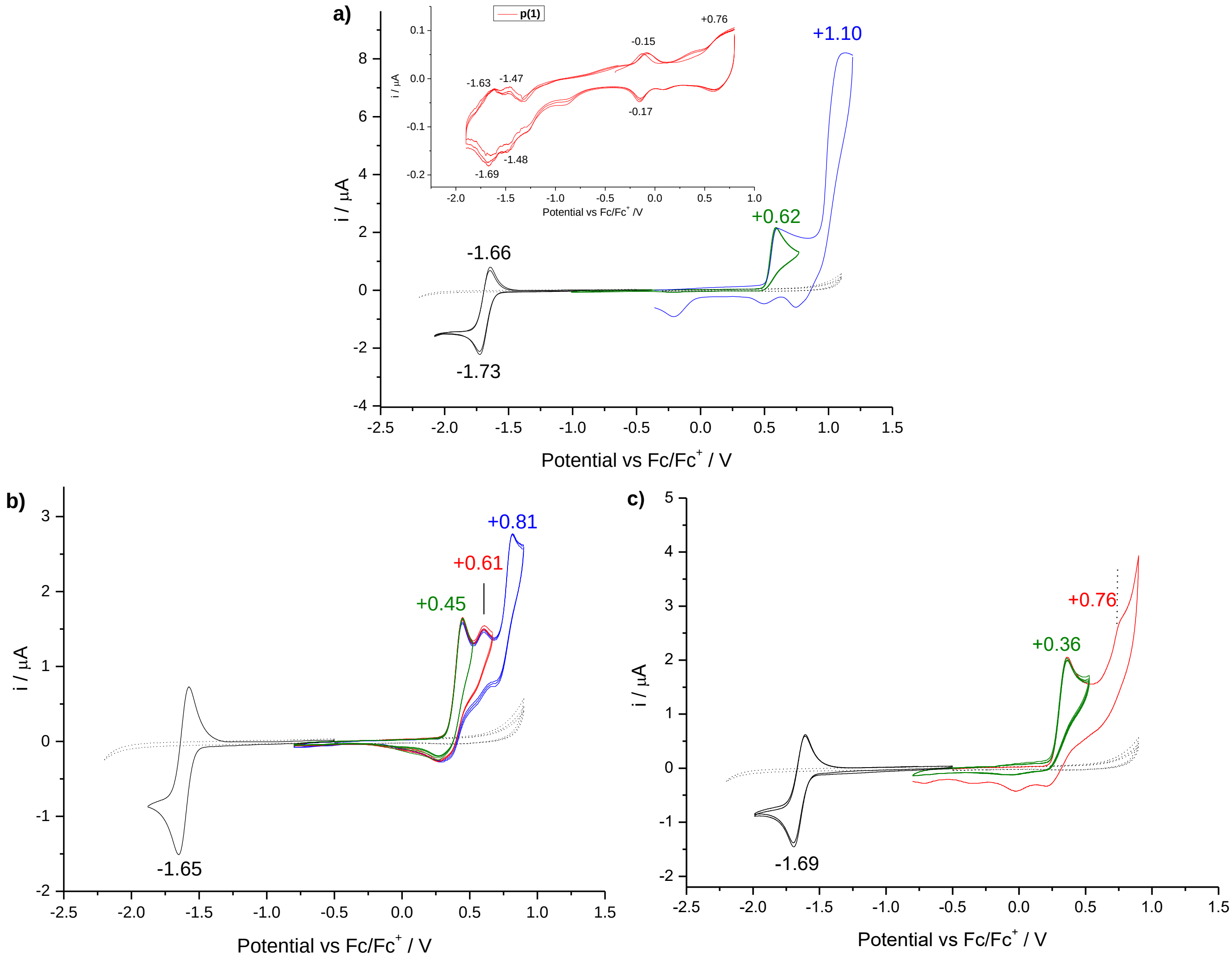

Figure S1. CV curves limited by first (*green*), second (*red*) and third oxidation (*blue*) and reduction peaks (*black*) registered in 1mM solution of **1** (**a**), **2** (**b**), **3** (**c**); in 0.1 M Bu_4NBF_4/DCM. **p(1)-I** deposited in the range −0.20–0.87 V while scanning between −1.89 and +0.87 V–(a) insert; 50 mV/s.

Table S6. Optimized structures of **1** π-dimers of radical cations (left) and di(radical cations) (right), calculated at ϖB97X-D functional combined with 6-31G(d,p) basis set.

**Π-dimer of radical cations**	**Π-dimer of di(radical cations)**
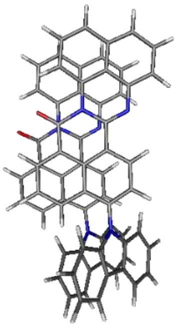	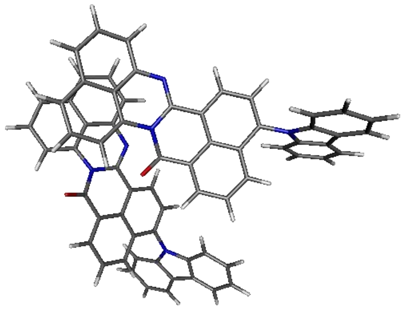
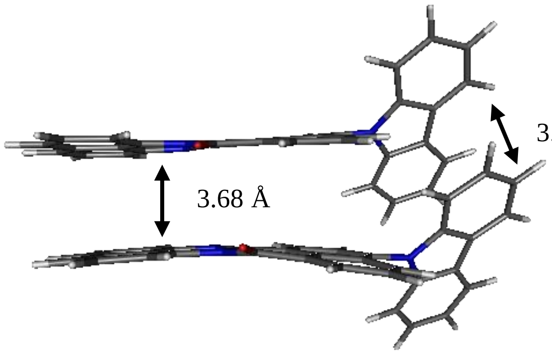	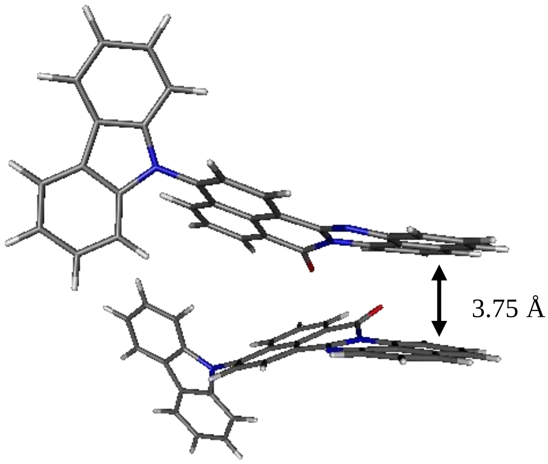

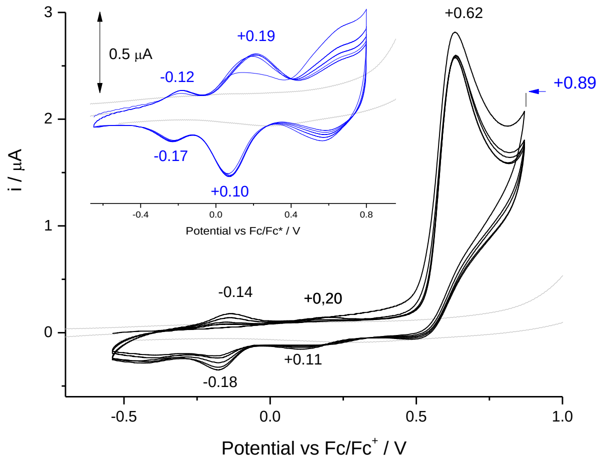

Figure S2. CVs limited after first oxidation peaks to +0.89 V in 5 mM of **1** and 0.1 M Bu_4NBF_4/DMSO; product under polarization in monomer-free 0.1 M Bu_4NBF_4/DMSO (insert); 50 mV/s.

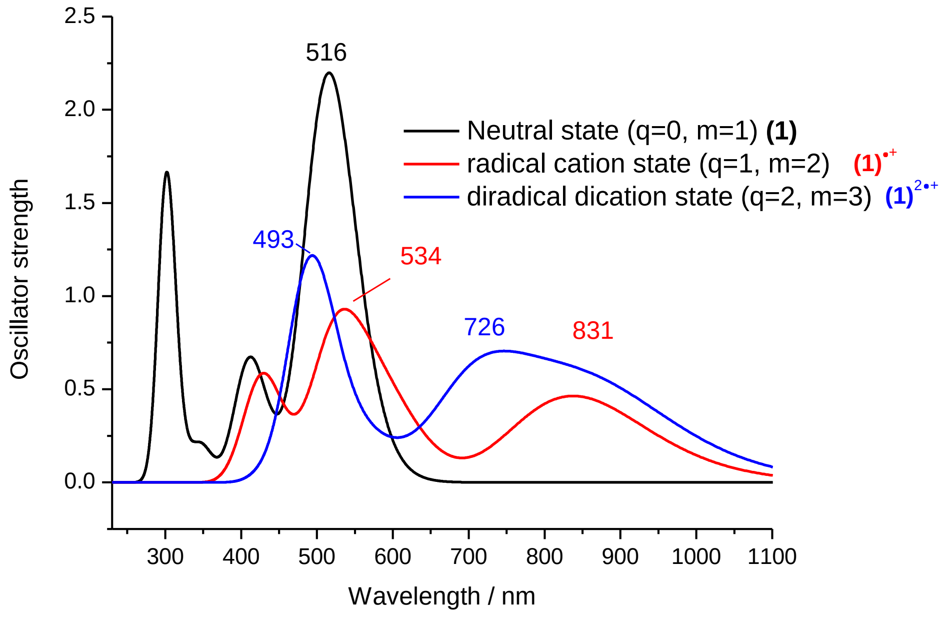

Figure S3. Simulated UV-Vis-NIR spectra of **1** states, calculated at TDDFT/CAM-B3LYP/6-31G(d)/CPCM(DCM).

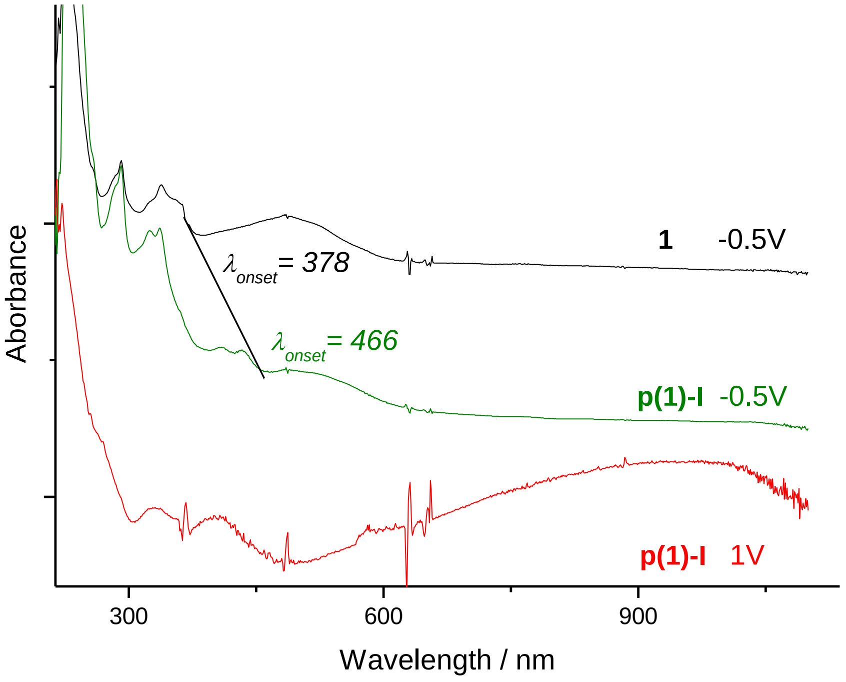

Figure S4. UV-Vis-NIR spectrum for neutral monomer **1** (*black*); neutral **p(1)-II** under −0.5 V polarization (*green*) ; **p(1)-II** oxidized at 1V (*red*) in 0.1 M Bu_4NBF_4/DCM solution.

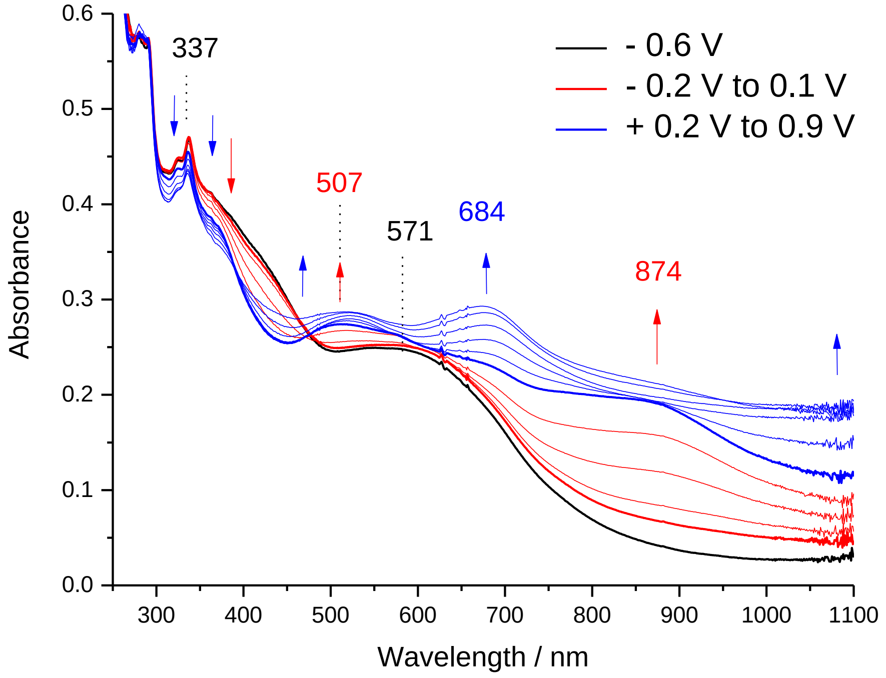

Figure S5. Change in absorbance of UV-Vis under polarization of **p(3)** in the range of polaron (*red*) and bipolaron forming (*blue*) in 0.1 M Bu_4NBF_4/DCM solution.
